# Bacterial iron reduction and biogenic mineral formation for the stabilisation of corroded iron objects

**DOI:** 10.1038/s41598-017-19020-3

**Published:** 2018-01-15

**Authors:** Wafa M. Kooli, Lucrezia Comensoli, Julien Maillard, Monica Albini, Arnaud Gelb, Pilar Junier, Edith Joseph

**Affiliations:** 1 Laboratory of Microbiology, Institute of Biology, University of Neuchâtel, 2000 Neuchâtel, Switzerland; 2 Laboratory of Technologies for Heritage Materials, Institute of Chemistry, University of Neuchâtel, 2000, Neuchâtel, Switzerland; 3 Laboratory for Environmental Biotechnology, ENAC-IIE-LBE, Ecole Polytechnique Fédérale de Lausanne, 1015 Lausanne, Switzerland; 4 Haute Ecole Arc Conservation-Restauration, HES-SO, 2000 Neuchâtel, Switzerland; 5 Present Address: Laboratory of Mechanical Systems Engineering, Swiss Federal Laboratories for Materials Science and Technology, 8600 Dübendorf, Switzerland

## Abstract

Exploiting bacterial metabolism for the stabilisation of corroded iron artefacts is a promising alternative to conventional conservation-restoration methods. Bacterial iron reduction coupled to biogenic mineral formation has been shown to promote the conversion of reactive into stable corrosion products that are integrated into the natural corrosion layer of the object. However, in order to stabilise iron corrosion, the formation of specific biogenic minerals is essential. In this study, we used the facultative anaerobe *Shewanella loihica* for the production of stable biogenic iron minerals under controlled chemical conditions. The biogenic formation of crystalline iron phosphates was observed after iron reduction in a solution containing Fe(III) citrate. When the same biological treatment was applied on corroded iron plates, a layer composed of iron phosphates and iron carbonates was formed. Surface and cross-section analyses demonstrated that these two stable corrosion products replaced 81% of the reactive corrosion layer after two weeks of treatment. Such results demonstrate the potential of a biological treatment in the development of a stabilisation method to preserve corroded iron objects.

## Introduction

Iron is an ubiquitous material. Since the industrial revolution, iron alloys (especially steel and cast) have been used at a large scale in fields as diverse as architecture, civil engineering, transport, food industry, and art^1^. Nowadays, iron is the most mined element in the world and constitutes 90% of all refined metals^1^. The widespread use of iron is explained by its metallurgical properties (ductility, hardness, strength)^2^. However, iron can be easily corroded by electrochemical reactions or by microbiologically influenced corrosion (MIC)^3,4^. Corrosion of metals leads to the modification of the exposed surface and the formation of a corrosion layer^5^. Depending on the environmental conditions, the corrosion products formed can protect the surface from further change (chemically stable corrosion products)^3,6,7^. Indeed, some studies have demonstrated that the presence of magnetite (Fe_3_O_4_), siderite (FeCO_3_) or vivianite (Fe_3_(PO_4_)·2H_2_O) plays a significant role in the preservation of archaeological iron objects and of iron water pipes^8,9^. These minerals are less reactive (especially to oxygen and humidity), and their formation leads to the preservation of the object^8^. In other cases, the corrosion products formed can cause degradation and irreversible damage (active corrosion)^1,10^. Examples of such active corrosion products include some Fe(III) oxyhydroxides, goethite α-FeO(OH), and lepidocrocite γ-FeO(OH)^11^. In particular, active corrosion is promoted by chloride ions that can be part of the formed corrosion products (for instance in akageneite β-FeO(OH)Cl)^10,12–15^. When the relative humidity is high, Fe(II) chloride salts absorb water vapor, dissolve, and form wet droplets of an orange colour causing a phenomenon described as weeping of iron^16^. Ultimately, if no conservation-restoration intervention is undertaken, these reactive corrosion products create physical stress and can lead to the integrity loss of the artefact^17^.

Iron corrosion causes considerable economic losses due to the costs of maintenance and replacement of damaged of iron items^9–18^. Therefore, limiting the formation of reactive corrosion products or converting those into more stable ones are two key issues for the preservation of iron surfaces. The former can be achieved by storage under anoxic conditions, increasing pH (passivation), or by applying coatings or anticorrosion agents. Chemical transformation of the reactive corrosion products promotes the latter. Given that the procedures used so far for limiting iron corrosion can be harmful, costly, time consuming, and also produce large amounts of waste^1,14,16–19^, there is a growing interest in developing green alternatives to traditional iron stabilisation methods^20^.

Using bacteria or bacterial enzymes to stabilise iron surfaces has been investigated previously^3^. For example, the iron reducing bacterium *Geobacter sulfurreducens* or a purified hydrogenase obtained from *Ralstonia eutropha* were used in separate studies to produce iron phosphates on steel^6,7^. Likewise, the incubation of mild steel in a culture inoculated with biofilm-forming *Rhodococcus* sp. strain C125 and *Pseudomonas putida* mt2 led to the formation of a protective surface layer of the mineral vivianite^21^. Recently a study with *Desulfitobacterium hafniense* showed that this bacterium can reduce reactive Fe(III) oxyhydroxides from corroded iron objects, favouring the biogenic production of vivianite^22^. However, abiotic reduction and undesirable formation of additional corrosion products were observed alongside biological iron reduction due to the composition of the growth medium. In addition, working with a strictly anaerobic bacterium such as *D. hafniense* could be challenging for the transfer of a biotechnological solution from the laboratory into real conservation praxis. Using a facultative anaerobe to perform iron reduction in a two-step procedure is an alternative to solve both of these issues. In the first step biomass can be produced aerobically in optimal growth conditions. After collecting the bacterial biomass and removing any carryovers from the culture medium used for aerobic growth, the concentrated cells are injected, in a second step, to an anaerobic chemical matrix. Under anaerobic conditions iron reduction can take place and crystalline mineral phases can be formed due to the interaction of reduced iron and the chemical precursors in the matrix. Handling of a facultative anaerobe under these conditions facilitates the transfer of technology into praxis. The overall hypothesis in this approach is that active biomass will still perform iron reduction with minimal growth requirements, inducing the formation of specific minerals as by-products of the interaction with the chemical precursors present in the matrix. This hypothesis was tested here. We evaluated iron reduction and biogenic iron mineral formation using *Shewanella loihica* strain PV-4, a halophilic facultative anaerobe that reduces iron and was involved in magnetite production^23,24^. Active biomass of *S. loihica* was obtained from aerobic batch cultures and used for the production of crystalline iron minerals in various chemical matrices. Finally, conditions leading to the formation of crystalline minerals were applied on corroded iron coupons to demonstrate the performance of the proposed treatment.

## Results

### Reduction of ferric citrate and ferric chloride and characterization of the minerals formed

*S. loihica* is known for its ability to grow using iron as electron acceptor for respiration and to form doped magnetite particles after 75 days of incubation^24^. We first investigated iron reduction in three chemically defined matrices using biomass produced under aerobic conditions. For this, we first grew *S. loihica* aerobically in LB medium overnight and collected the biomass. Biomass (10^8^ cells for 20 mL) was washed to avoid any carry over of the spent growth medium and then transferred to anaerobic bottles containing three different anoxic chemical matrices. These chemical matrices were designed to favour the production of iron oxides (IOx), iron carbonates (ICarb), or iron sulphides (ISulp) as biogenic minerals. Ferric citrate (Fe citrate) or FeCl_3_ were used as iron sources. Controls were performed using the same chemical matrices in the absence of bacteria. Iron reduction was monitored for 24 h by measuring the concentration of Fe(II) in solution. The increase of Fe(II) in the bottles containing the bacteria confirmed that *S. loihica* could reduce the two iron sources (SI-1).

**Figure 1:**
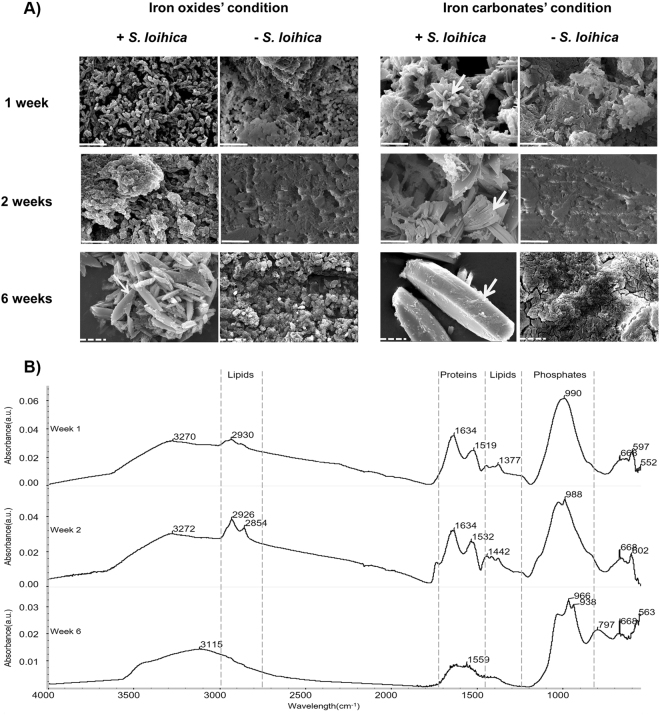
Soluble Fe(III) reduction and biogenic mineral formation. (**A**) Scanning electron microscopy (SEM) images of samples collected during iron reduction in Fe citrate-containing iron oxides (IOx) and carbonate (ICarb) matrices in presence (+*S. loihica*) or absence (−*S. loihica*) of *Shewanella loihica*. The results show the formation of a solid structured mineral phase after 1, 2, and 6 weeks of incubation. The white arrows indicate the sites where elemental analysis was performed. Continuous and dashed white scale lines correspond respectively to 5 µm and 10 µm. (**B)** ATR-FTIR spectra of culture pellets from iron-reducing ICarb solution contaning Fe citrate after 1, 2 and 6 weeks of incubation. The spectral region corresponding to the absorbance peaks of lipids, proteins, and phosphates are marked out by dashed lines.

Biogenic mineral formation was assessed after one, two, and six weeks of incubation. In general, a change in the colour of the solutions incubated with bacteria was observed in comparison to the abiotic controls (SI-2). In addition, in presence of bacteria, the formation of solid structured mineral phases was observed in the IOx (after six weeks) and ICarb (visible from one week onwards) matrices containing Fe citrate (Fig. 1A). The size of the mineral particles increased overtime in the ICarb solution (Fig. 1A). Elemental analyses (Energy Dispersive Spectroscopy-EDS) of the mineral particles indicated that they are composed of iron, oxygen, carbon, and phosphorus (Table 1). The latter was only detected when a precipitate was observed (Fig. 1A, SI-2, and SI-3). In the controls without bacteria the composition of the particles observed after lyophilisation suggested only the precipitation of the salts present in the medium (with Na and Cl among the major elements detected; SI-3). The presence of phosphorus was unexpected, as no phosphate was added to the chemical matrices for performing iron reduction. In addition, this element was not detected when solid structured phases were absent (SI-3). Since the biomass was washed prior to the transfer into the iron reduction matrices, is unlikely that phosphate originates from spent medium used during the step of production of the biomass.

**Table 1:** Elemental composition in terms of atomic percentage (AT%) obtained using energy-dispersive X-ray spectroscopy (EDS) *for the treatments* using Fe citrate with *S. loihica* (+), which resulted in the formation of biogenic minerals (ICarb and IOx conditions).

Elements (AT%)	ICarb condition			IOx condition
	1 week	2 weeks	6 weeks	6 weeks
Fe	15.37	15.26	19.18	15.61
O	24.00	33.66	46.42	51.02
C	33.74	26.64	19.22	20.75
P	5.60	9.89	12.77	11.23
Trace elements	21.29	14.55	2.41	1.39

Fourier Transform Infrared Spectroscopy (FTIR) analyses were performed to identify the minerals formed. The results obtained with the IOx (SI-4) and ICarb chemical matrices were comparable (Fig. 1B). After one and two weeks of incubation typical vibration bands for proteins (amide I peak around 1640 cm^−1^ and amide II peak around 1520 cm^−1^) and lipids (C-H stretching around 2930, 2854 cm^−1^ and binding peaks at 1397 cm^−1^) were observed, indicating the presence of bacteria. The peaks comprised between 1040 and 940 cm^−1^ correspond to the phosphate absorbance region^25,26^, which confirmed the elemental analyses, and thus the formation of iron phosphates. Also, according to the differences observed in the spectra collected overtime, iron phosphates changed from an amorphous to a more crystalline phase. In amorphous compounds, water is not as coordinated as in crystalline forms. Absorbance bands corresponding to the coordinated water associated with iron phosphates can be observed at 3447 and 797 cm^−1^ only after six weeks of incubation. According to the obtained FTIR spectra the biogenic minerals belong to the family of vivianites or barbosalites^25,26^.

### Iron reduction with corroded iron plates and characterization of the minerals formed

As the aim of the study was the production of stable iron minerals by the conversion of reactive corrosion products, we performed further experiments with corroded iron plates using exclusively the ICarb chemical matrix, which clearly favoured crystalline mineral formation in the previous experiment. The iron source was in this case the corrosion products (mainly goethite and lepidocrocite; SI-5) formed on an iron plate corroded in a marine atmosphere. The plates were sterilised and added under anoxic conditions to anaerobic bottles containing the ICarb sterile matrix. Biomass of *S. loihica* was prepared and inoculated as described before (10^8^ cells in 20 mL).

**Figure 2:**
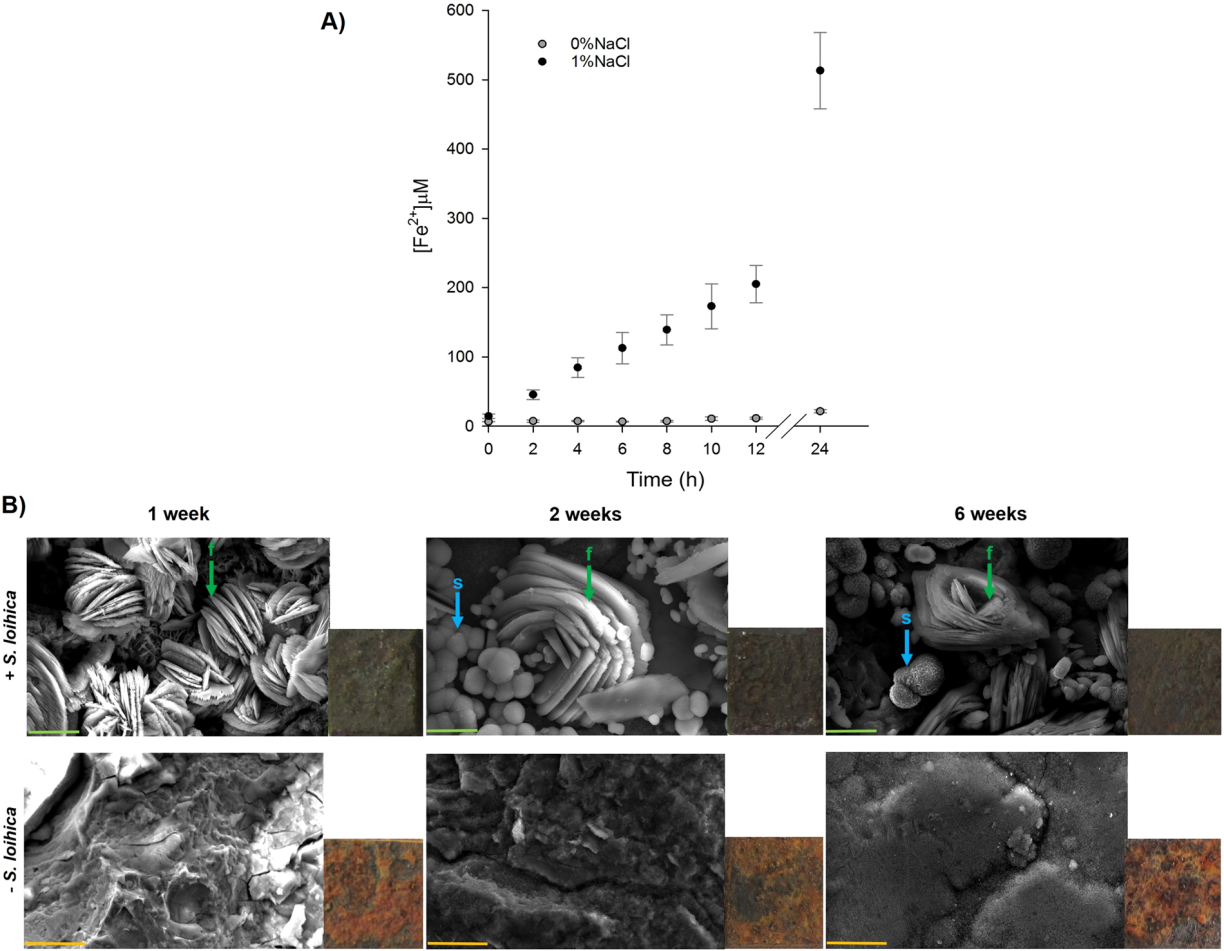
Solid Fe(III) reduction and biogenic mineral formation. (**A**) Iron reduction in cultures of *S. loihica* without (grey circles) and with 1% NaCl (black circles). (**B**) Scanning electron microscopy (SEM) images and visual aspect of the iron coupons treated with *S. loihica* (+*S. loihica*) and of the abiotic control (−*S. loihica*) in the presence of 1% NaCl, after 1, 2, and 6 weeks of incubation. The letters f and s indicate foil-like biogenic mineral habita and spherical agregates respectively. Scale bars: orange: 20 µm and green: 10 µm.

*S. loihica* is a halophile that requires the presence of salt for growth. We tested growth at different concentrations of NaCl (SI-6) and with different sodium sources (SI-7), confirming that 2% NaCl is the optimal salt concentration for bacterial growth. The addition of a salt containing chlorides to the reductive chemical matrix is not ideal given the deleterious role of chloride ions as active iron corrosion instigators. Therefore, we tested iron reduction without NaCl (0%) and 1% added NaCl on corroded iron coupons. In the treatment without NaCl we wanted to test if the salts potentially contained in the natural corrosion patina (i.e. salts deposited from aerosols produced by the marine environment in which the coupons were corroded) were enough to meet the salt requirements of a halophilic strain, such as *S. loihica*. Iron reduction using corroded coupons as iron source was assessed by the increase of Fe(II) concentration in the solution. The results showed that *S. loihica* was only able to reduce solid Fe(III) when 1% NaCl was added (Fig. 2A). Therefore salts present in the corrosion layer of the iron plates do not provide the required salt concentration for the metabolic activity of *S. loihica*. Without NaCl, no reduction was observed although the bacterial cells remained intact when observed under the microscope. The fact that iron reduction did not occur at 0% NaCl provided an ideal inactive biological control to establish the effect of the bacterial treatment (at 1% NaCl) on the conversion of the corrosion layer. As expected, no mineral formation was observed for the treatment with bacteria at 0% NaCl (SI-8). On the contrary, with 1% NaCl, SEM observations of the coupons’ surface confirmed the presence of a crystalline mineral phase already after one week of incubation (Fig. 2B). The production of minerals occurred alongside a change in the colour of the iron plates, from red to dark grey (Fig. 2B, SI-8B). EDS analyses showed the presence of phosphorus associated to the production of crystalline mineral phases (Table 2). Phosphorus was absent from the plates incubated in the abiotic medium (Table 2; *−S. loihica*). Likewise, phosphorus was not detected by EDS analyses on the coupons treated at 0% NaCl, with and without the bacteria, as well as in the untreated coupons (SI-9).

**Table 2:** Elemental composition in terms of atomic percentage (AT%) obtained using energy-dispersive X-ray spectroscopy **(**EDS) for the minerals obtained in the treatment with 1% NaCl with (+) and without (−) *S. loihica*. “f” corresponds to the foil-like aggregates, while “s” refers to the spherical-shaped minerals. nd stands for “not detected”.

Elements (AT%)	1 week		2 weeks			6 weeks		
	+	−	+		−	+		−
			f	s		f	s	
Fe	20.45	33.10	26.31	24.76	43.69	22.88	21.48	44.25
O	50.69	65.41	38.36	50.67	54.89	51.09	51.79	54.35
C	15.37	nd	23.09	24.57	nd	10.65	26.73	nd
P	12.49	nd	12.25	nd	nd	15.38	nd	nd
Cl	nd	0.37	nd	nd	nd	nd	nd	nd
Trace elements	1.00	1.12	0.00	0.00	1.42	0.00	0.00	1.4

On the coupons treated with bacteria at 1% NaCl, two types of mineral forms were observed simultaneously after two and six weeks of incubation: foil-like habita (indicated as “f” in Fig. 2B and visible already at 1 week) and spherical aggregates (indicated as “s” in Fig. 2B). The EDS spectra of the two mineral phases differed. While the presence of phosphorus was a chemical signature of the foil-like habita (Table 2, f), the spherical-shaped aggregates contained only Fe, C, and O, but no traces of phosphorus (Table 2, s). As in the case of the soluble iron sources, the presence of phosphorus in the foil-like habita minerals was surprising given that no P source was added to the reductive chemical matrix. It is known that some bacteria are able to accumulate phosphate inside the cells in the form of polyphosphates. Depending on the metabolic state of the bacterial cells, phosphorus can later be released in the form of orthophosphates in the solution^27,28^. DAPI staining and microscopic observations of *S. loihica* showed that this bacterium is indeed able to accumulate polyphosphates in granules in oxic conditions, releasing orthophosphates in anoxic conditions during the treatment (SI-10). These orthophosphates are a likely P source for the biogenic formation of iron phosphates.

### Effect of biogenic mineral formation on the corrosion layer of iron coupons

**Figure 3:**
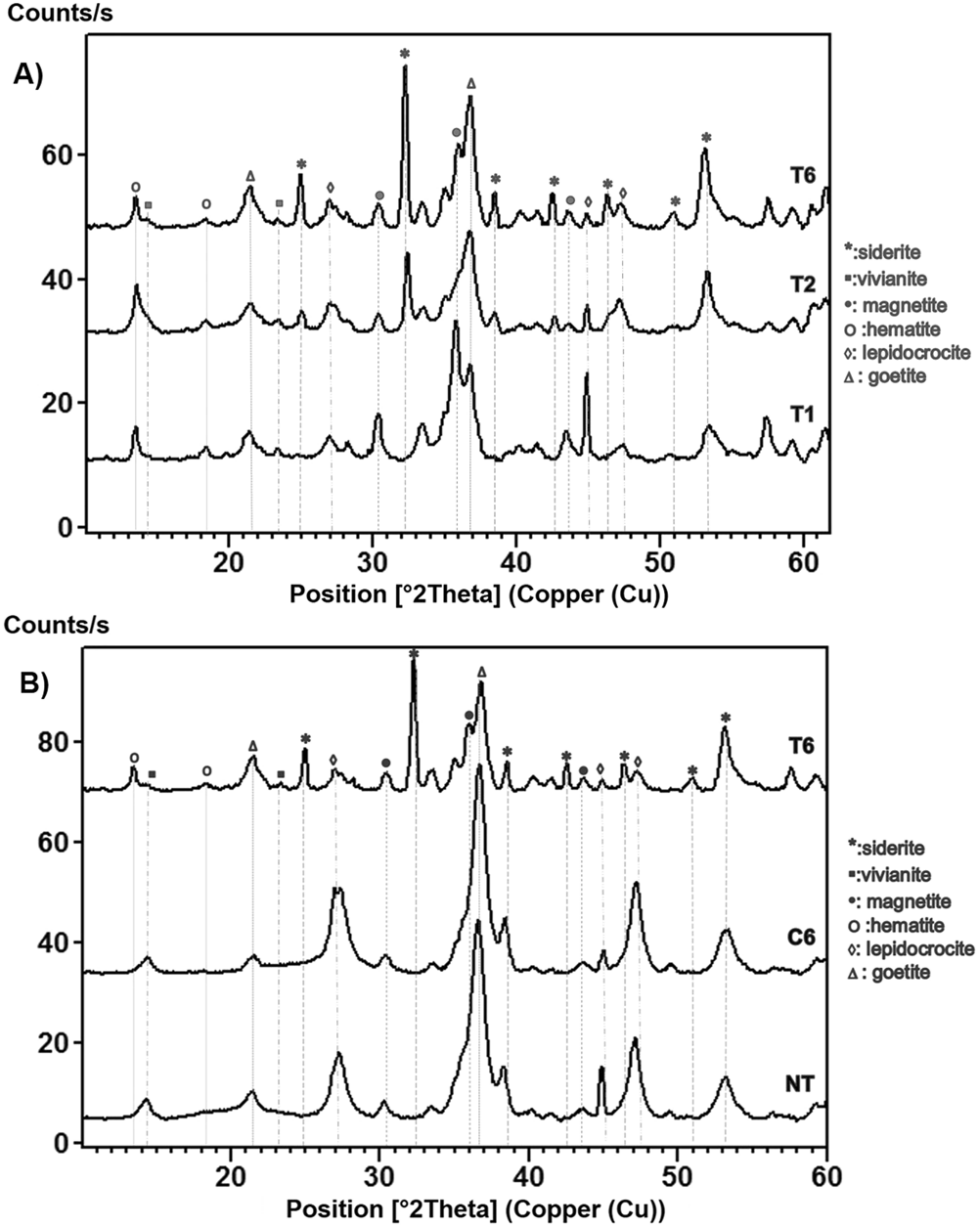
X-Ray diffraction (XRD) spectra of iron coupons. (**A**) Spectra recorded on the coupons treated with *S. loihica* after 1 (T1), 2 (T2) and 6 (T6) weeks of incubation. (**B**) Spectra recorded after 6 weeks of incubation of the coupons treated with *S. loihica* (T6), the abiotic control (C6), and the untreated coupons (NT).

Mineral identification before and after the bacterial treatment of iron coupons was conducted using XRD analysis. The results were consistent with the EDS analyses, showing the presence of a Fe(II) phosphate mineral (vivianite), already after one week of incubation (Fig. 3A). In addition, Fe(II) carbonate (siderite; FeCO_3_), was detected on the treated coupons after two and six weeks of incubation (Fig. 3A). The XRD results also demonstrated the absence of Fe(II) phosphates or Fe(II) carbonates in the abiotic controls and on the untreated coupons (Fig. 3B). Moreover, the intensity of the XRD peaks related to reactive corrosion products (e.g. hematite and lepidocrocite) decreased over time in coupons treated with bacteria at 1% NaCl (Fig. 3B). This result clearly demonstrated the effectiveness of the biological treatment to replace reactive corrosion products by stable biogenic minerals.

**Figure 4:**
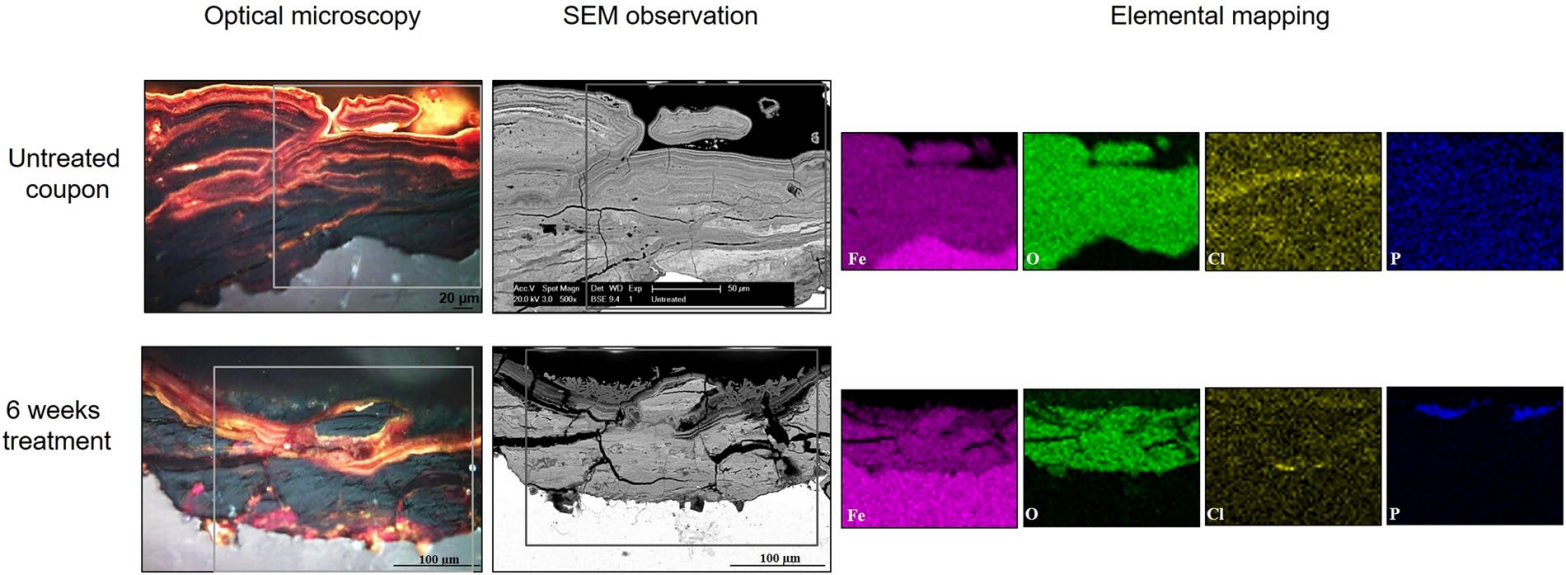
Cross-section analysis of the corrosion layer in iron coupons. Optical microscopy and scanning electron microscopy (SEM) images of the untreated and treated coupons after 6 weeks of incubation. The area where elemental mapping was performed is indicated by a grey box. Elemental mapping showing the presence of iron (pink), oxygen (green), chlorine (yellow), and phosphorus (blue).

**Figure 5:**
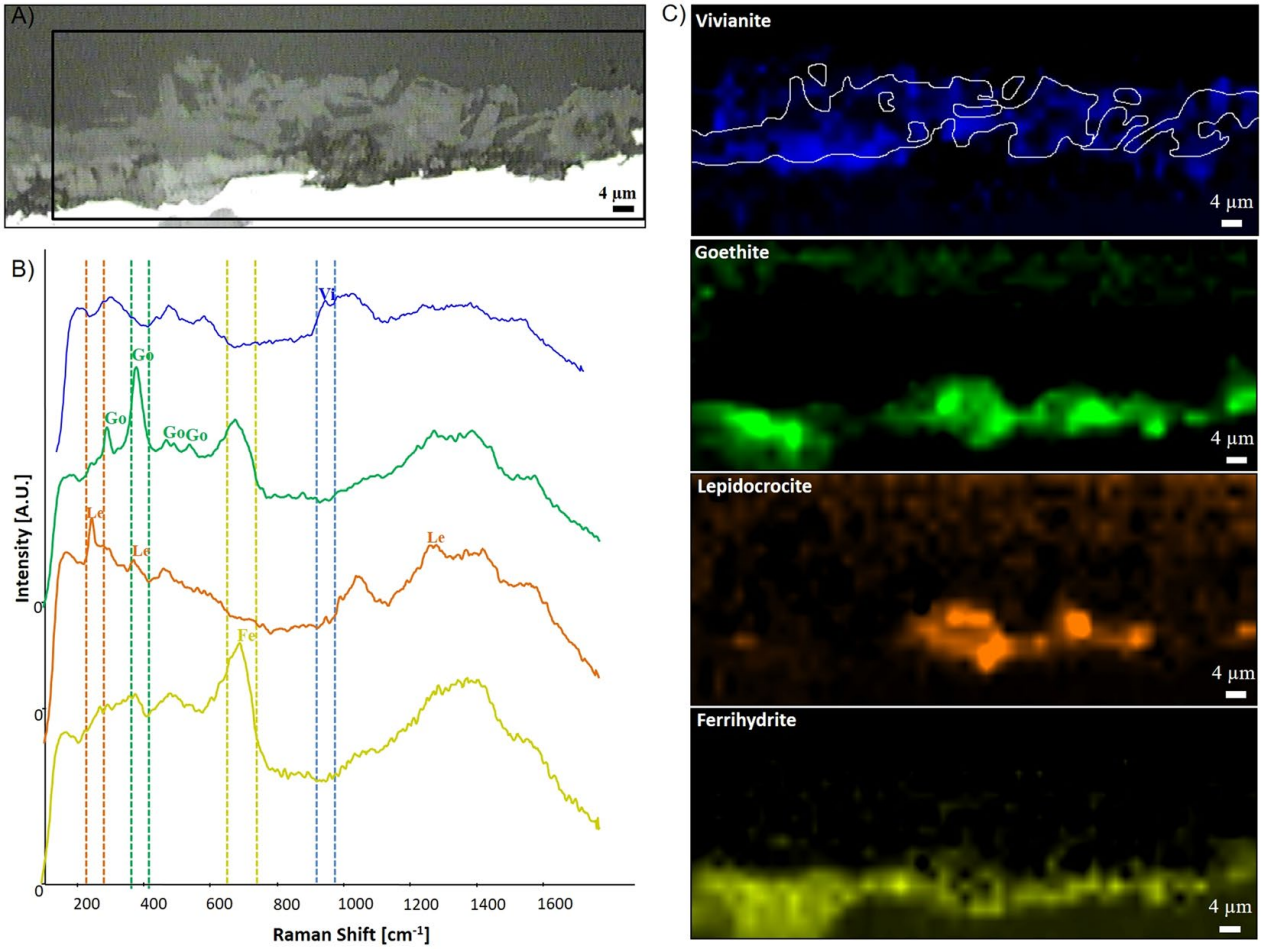
Raman mapping of a bacterial-treated coupon after 1 week of incubation. (**A**) Scanning electron microscopy image with the area analysed by Raman mapping indicated by a black box. (**B**) Raman spectra of vivianite (Vi), goethite (Go), lepidocrocite (Le), and ferrihydrite (Fe), extracted from the map dataset and indicating the vibrational bands used for the elaboration of the chemical maps. (**C**) Raman chemical maps of vivianite (964 cm^−1^) in blue, of goethite (384 cm^−1^) in green, of lepidocrocite (248 cm^−1^) in orange, and of ferrihydrite (705 cm^−1^) in yellow.

In addition to the surface analyses, cross-section analyses were performed. Untreated iron coupons displayed a corrosion layer with dark-brown, red and orange tonalities (Fig. 4, SI-11). The same was observed for the abiotic control coupons (SI-11 and SI-12). In contrast, a precipitation of grey-green minerals was observed on the outermost corrosion layer of the coupons treated with bacteria (SI-11). An overall decrease on the thickness of the original corrosion layer was measured after the treatment with bacteria. The continuity of the biogenic layer was estimated and the results confirmed that bacteria produced a relatively homogeneous mineral layer on top of the original corrosion layer covering already 81% of the coupons’ surface after 2 weeks of incubation (SI-11). SEM observations of the cross-sections after six weeks of treatment revealed that untreated coupons (Fig. 4) and abiotically treated coupons (SI-12) had a corrosion layer mainly composed of iron, oxygen and chlorine. In contrast in the coupons treated with bacteria, Fe and O were found to be the main components of the corrosion layer (Fig. 4). Additional molecular analyses carried out on the cross-sectioned coupons after 1 week of treatment confirmed that the biogenic layer was mainly composed of vivianite with the presence of a characteristic Raman shift at 964 cm^−1^ (Fig. 5). Below this layer, the same corrosion products detected on the untreated coupons were also located and identified as goethite, lepidocrocite, and ferrihydrite (Fig. 5). No chlorinated compounds such as akaganeite β-FeO(OH)Cl were individuated by Raman investigation, neither on the untreated nor on the treated coupons. This is probably due to the concomitant presence of ferrihydrite whose Raman shifts superimpose those of akageneite.

## Discussion

The aim of the proposed biological treatment to stabilise corroded iron is the production of specific iron minerals by the conversion of reactive corrosion products. Minerals such magnetite or siderite are desirable because they have a colour similar to the native colour of iron (black-grey). Moreover, these minerals are reported to be thermodynamically stable and are naturally found on iron objects acting as protective layer^8,11,16,29,30^. In this study, we developed a two-step approach based on the aerobic production of bacterial biomass followed by the anaerobic reduction of iron for the formation of specific biogenic minerals. Buffering the medium at the optimal pH reported for *S. loihica* (pH 6.5–7) precluded the formation of magnetite^24,31^. Also, recent studies have shown that under specific conditions (e.g. H_2_ as electron donor) magnetite could be destabilised by iron reducing bacteria^32,33^ and hence its formation may not be desirable. In the case of the chemical matrix to favour the production of iron sulphides, the formation of an amorphous phase of FeS instead of a crystalline one is probably the result of an insufficient concentration of dissolved sulphides^31^. The condition favouring the production of iron carbonates was suitable for biogenic mineral formation. However, in addition to the production of siderite after prolonged incubation (2 weeks onwards), vivianite was also precipitated. Vivianite (solubility constant −36.00^34^) is expected to be the initial Fe(II) phase to precipitate in a phosphorus-containing medium at a near neutral pH^8^. Experiments performed by Zachara *et al*.^35^ confirmed that in presence of 4 mM phosphates the formation of vivianite precedes the one of siderite (solubility constant −10.68)^34,36^, and that the latter precipitates only after the complete incorporation of phosphorus in vivianite^35^. Vivianite and siderite are biogenic by-product minerals of dissimilatory Fe(III) reduction in bicarbonate buffers^31,35,37,38^. For instance, *Rhodococcu*s sp. C125 and *Pseudomonas putida* mt2 are able to induce vivianite formation on mild steel coupons in a phosphates-containing solution^39^. However, the production of iron phosphates under the treatment conditions used here was unexpected given the lack of added phosphorus in the reductive chemical matrix. Although dead bacterial biomass and/or the release of proteins containing phosphate groups as well as degradation of nucleic acids^40^ are a possible source of phosphorus, accumulation of polyphosphates by *S. loihica* during aerobic growth and release of orthophosphates during the treatment under anoxic conditions is a more likely explanation as to the source of phosphorus for vivianite precipitation.

Production of vivianite has been proposed as an anti-corrosion treatment for steel^39^. Therefore, according to the results obtained, the proposed biological treatment seems to be a good alternative to stabilise corrosion of iron objects. Analyses performed on the corrosion layer of iron coupons demonstrated that the biological treatment led to the decrease of reactive corrosion products along with an increase in the formation of biogenic minerals that are integrated in the natural corrosion layer. This was accompanied by a pleasing aesthetical change in the colour of the coupons to grey after 2 weeks of incubation. In addition, chlorides appeared to be almost completely extracted from the corrosion layer, avoiding any further harmful cyclic and active corrosion to start again. The biogenic minerals produced using *S. loihica*, vivianite and siderite, are reported to have a protective effect on water distribution pipes and archaeological iron objects^8,12^. Moreover, using a facultative anaerobe and the production of biomass under aerobic conditions facilitates the practical use of bacteria, compared to strict anaerobes^22^. Next, scaling up of the process and assessing the stability of the biogenic minerals produced and their long-term protective effect on corroded iron surfaces are required for the application of this stabilisation method into real conservation-restoration praxis. Nevertheless, exploiting bacterial metabolic activity to stabilise corroded iron objects is an innovative biotechnological process that can have a significantly positive impact on the preservation and maintenance of iron-based surfaces.

## Material and Methods

### Bacterial strain and aerobic growth conditions

*Shewanella loihica* PV-4 (DSM 17748) was used in this study. The strain was purchased from the DSMZ (Deutsche Sammlung von Mikroorganismen und Zellkulturen GmbH,). Regular cultivation was performed aerobically using Luria-Bertani medium (LB; 1% (w/v) tryptone, 0.5% (w/v) yeast extract, and 1% (w/v) sodium chloride (NaCl)) at pH 7 and at 20 °C under agitation at 130 rpm. For the experiments the bacterium was cultivated aerobically overnight. Cell biomass was then collected by centrifugation (1700 × *g* for 10 min) and the pellet was washed three times with an oxic basal solution containing 20 mM 1,4-Piperazinediethanesulfonic acid (PIPES) and the same (w/v) concentration of NaCl of the reducing matrix (see below) to avoid the carryover of spent LB medium. Between the washing steps the cells were centrifuged at 1700 × g for 10 min, discarding the supernatant at every step. Finally the pellet was resuspended in the same solution without NaCl and adjusted to a density of 10^8^ cells/mL to be added to the defined chemical matrices containing iron (SI-13).

### Reduction of ferric citrate and ferric chloride

Three chemical matrices were used for iron reduction using ferric citrate and ferric chloride as electron acceptors. For this, sodium lactate (final concentration of 5 mM) as electron donor, and ferric citrate (Fe citrate) or ferric chloride (FeCl_3_) (final concentrations of 10 mM) as electron acceptors were added to the same basal solution (20 mM PIPES, 2% (w/v) NaCl). For the production of specific iron minerals, sodium hydroxide (NaOH) or sodium bicarbonate (NaHCO_3_) (final concentrations of 20 and 50 mM, respectively), were added to favour iron oxides (e.g. magnetite; IOx) or (e.g. siderite; ICarb), respectively. A third treatment consisted of 20 mM NaOH supplemented with 10 mM sodium sulphide (Na_2_S) to induce the production of iron sulphides (ISulp) (SI-13). In the case of ISulp, after assessing iron reduction for 24 h in the (IOx) matrix, sodium sulphide was added to favour iron sulphide mineral production. The final pH of the three matrices was between 6.5 and 7 (optimal pH for *S. loihica*). To render the iron reduction matrices anoxic, the solutions were first boiled to remove dissolved oxygen and then flushed with nitrogen. The FeCl_3_ stock solution (final concentration 0.5 M) was filter sterilized under anoxic conditions, because we have observed the formation of precipitates if the solution was sterilized by autoclaving. All the other solutions were sterilized by autoclaving at 120 °C for 20 min. The NaHCO_3_ solution was added after autoclaving to avoid CO_2_ degassing. Biomass was prepared as described above and added to the reducing matrix to a final concentration of 5 × 10^6^ cells/mL. To evaluate iron reduction, 900 µL samples were collected every 2 h for 24 h to measure the concentration of Fe(II) in solution (see below). The production of iron minerals was evaluated after 1, 2, and 6 weeks of incubation at room temperature under agitation (120 rpm). Experiments were performed in triplicates including abiotic controls. In the latter the same defined matrices were incubated in the absence of bacteria to investigate abiotic iron reduction and chemical mineral formation.

### Reduction of corroded iron plates

For the validation of the treatment on real objects, we used iron plates (50 × 50 × 2–3 mm) naturally corroded after one-year exposure in an outdoor marine environment containing chlorinated aerosols (French Institute of Corrosion, Brest, France). The orientation and position of the iron plates during exposure were selected according to ISO 9223 standard, which defines the standards for the corrosion of metals and alloys. Plates were exposed south skyward at 45° from the horizontal. For the experiment, the plates were then cut in coupons of 10 × 10 × 2–3 mm. The preparation of the plates for the experiments was performed under oxic conditions.

Iron reduction and biogenic mineral formation with the corroded iron coupons was performed in a matrix composed of 20 mM PIPES, 5 mM sodium lactate, and 50 mM NaHCO_3_. In this case, two conditions were compared: no NaCl (0%) and 1% NaCl. The final pH of the matrices was between 6.6 and 7.4. For the sterilization of the iron coupons, autoclaving was avoided as an enhancement of the corrosion layer was observed under both oxic and anoxic conditions. Instead of autoclaving, coupons were washed with 70% (v/v) ethanol and dried under UV exposure for 1 h for each side. A control for this sterilization method was performed by incubating UV-sterilized plates on solid LB medium under oxic and anoxic conditions, which showed no bacterial growth after one week of incubation.

Biomass was prepared as described above and added to the reducing matrix to a final concentration of 5.10^6^ cells/mL. To evaluate iron reduction, Fe(II) was measured in 900 µL samples collected every 2 h for 24 h (see below). The production of iron minerals was evaluated after 1, 2, and, 6 weeks of incubation at room temperature under agitation (120 rpm). Experiments were performed in triplicates including abiotic controls (without bacteria).

### Measurements of Fe(II) concentration

To measure the reduction of Fe(III) into Fe(II), the ferrozine assay was performed following a modified protocol from Bell *et al*.^41^. First, 100 µL of 5 M HCl were added immediately after sampling to the 900 µL of collected samples and stored at 4 °C. The samples were centrifuged for 1 min at 6700 × *g*, then 100 µL of the supernatant were taken and 900 µL of ferrozine solution (0.1% ferrozine in a 100 mM HEPES solution at pH 7) were added. Fe(II) concentration was determined by measuring the absorbance at 562 nm with a UV-Vis spectrophotometer (Thermo Scientific Genesis 10 s). A calibration curve was obtained using serial dilutions of 1 mM ferrous ammonium sulphate solution in acidic MilliQ water (pH 2).

### Scanning Electron Microscopy coupled with Energy Dispersive X-Ray Spectroscopy (SEM-EDS) analyses

Samples from Fe citrate and FeCl_3_ amended cultures were centrifuged and fixed for 1 h with 2.5% glutaraldehyde solution in 0.1 M sodium cacodylate, washed twice by centrifugation with the same solution and fixed again for 2 h with 1% osmium tetraoxide in 0.1 M sodium cacodylate. After the secondary fixation, the samples were washed twice with distilled water. Then dehydration steps were performed using different concentrations of ethanol (25%, 50%, 75%, 90%, 100% (v/v)) and then pure acetone following a modified protocol from Pearson *et al*.^42^. Finally, samples were mounted on stubs using carbon conductive tape and coated with a 23 nm layer of gold using a Bal-Tec sputter coater SCD 005 (60 mA current, distance of the gold pastille to the sample: 5 cm, 60 s duration, argon gas). As there were no precipitates in the abiotic controls with Fe citrate, samples were lyophilised prior to stub mounting. The iron coupons were washed twice with deionised water and ethanol 70% (v/v) before being mounted on stubs using carbon conductive tape without gold sputtering. To avoid humidification, the stubs were stored in desiccators with silica gel. A Philips ESEM XL30 FEG environmental scanning electron microscope equipped with an energy-dispersive X-ray analyser was used. The samples and coupons were observed in secondary electrons mode at an acceleration potential of 10–25 keV and with a 10 mm working distance.

### Fourier Transform Infrared Spectroscopy (FTIR) analyses

FTIR measurements were performed on the same samples used for SEM-EDS analyses. An iS5 Thermo Scientific spectrometer with a diamond Attenuated Total Reflectance (ATR) crystal plate (iD5™ ATR accessory) was used. All spectra were acquired in the range 4000–650 cm^−1^, at a spectral resolution of 4 cm^−1^. A total of 32 scans were recorded and the resulting interferograms averaged. Data collection and post-run processing were carried out using Omnic™ software.

### X-Ray Diffraction crystallography (XRD) analyses

XRD analyses were performed on untreated, abiotic control and bacterially-treated iron coupons. The preparation of the iron coupons was the same as for SEM-EDS analyses. High-statistics θ−2θ (bulk) measurements were performed with a PANalytical X’Pert Pro MPD diffractometer equipped with a fast-linear detector and sample spinner. The measurement time was 60 h to acquire high statistics for better signal-to-noise ratio. The sensitivity of the XRD phase analyses was about 1% weight. ICSD codes were used for the identification of siderite (98–004–6078), vivianite (98-004-6142), magnetite (98-001-1782), hematite (98-001-2733), lepidocrocite (98-000-0843) and goethite (98-001-7328).

### Cross-section analyses

One sample for each condition tested (untreated, abiotic control and bacterial treatment) was embedded in methacrylate resin employing the EpoFix Kit (resin and hardener, Struers). Cross-polishing was performed with silicon carbide abrasive paper with 250, 500, and 1000 grit and Micro-Mesh abrasive cloths 1800, 2400, 3200, 3600, 4000, 6000, 8000, and 12000 grades. Microscopic observations on the cross-sectioned samples were carried out with a Polyvar MET optical microscope and microphotographs were collected with Axio Vision LE software. An estimation of the percentage of original corrosion layer covered by the biogenic crystals was extrapolated from the microscopic images. Using the working conditions indicated above, SEM-EDS mapping was carried out to ascertain the distribution and the elemental composition of the newly formed biogenic minerals. Non-destructive Raman spectroscopy was performed directly on the cross-sectioned samples to define the molecular composition of the corrosion layer before and after bacterial treatment. The analysis was carried out with a Horiba-Jobin Yvon Labram Aramis microscope equipped with a Nd:YAG laser of 532 nm at a power lower than 1 mW. The spectral interval analysed was between 100 and 1600 cm^−1^. Single points analyses were carried out with the following conditions: 400-µm hole, 200-µm slit, and 10 accumulations of 10 s. Raman mapping was performed in selected areas of cross-sectioned samples with a step size of 2.5 µm in x and y directions. The spectra recorded were corrected (automatic baseline correction) using LabSpec NGS spectral software. Reference spectra were used for identifying the compounds present and for elaborating chemical maps.

### Polyphosphate staining with 4’,6-diamidino-2-phenylindole (DAPI)

Polyphosphate detection was performed in bacterial cultures cultivated overnight in LB medium. A washing step with 1× Phosphate-buffered saline (PBS buffer; 137 mM NaCl, 10 mM Na_2_HPO_4_, 2.7 mM KCl, 1.8 mM KH_2_PO_4_, pH 7.4) buffer was done twice in order to remove the media components. One mL from a bacterial culture of 1 OD unit (at 600 nm) was centrifuged, the pellet was collected, washed 2 times with 1 mL of ice-cold 1× PBS and resuspended in 1 mL of ice-cold 4% (w/v) paraformaldehyde (PFA). The sample was incubated for 1 h at 4 °C. The pellet was then collected by centrifugation and washed three times with 1× PBS. This washing step was repeated twice in order to remove residual PFA that would produce auto-fluorescent particles. For DAPI staining, a solution of 50 µg/mL DAPI was used. Cells were placed onto a microscope slide and left to dry at room temperature. Cells were then covered with the DAPI solution and incubated in the dark at room temperature for 30 min. The slides were washed by dipping into distilled water and then air-dried. An anti-fading agent (Citifluor AF2) was applied on the slides before placing the cover slip. Observations were done with an epi-fluorescence microscope Zeiss Axioplan2 imaging, using UV excitation light. Excitation wavelength was 350 nm. DNA-DAPI combination results in a blue colour (maximum fluorescence emission at around 450 nm) while polyphosphate-DAPI complex has a bright yellow colour (emission at around 550 nm)^43^.

### Quantification of orthophosphate (orthoP) release using malachite green

The orthoP released by the cells was measured every hour during 24 h following the same procedure of sample collection as for the iron reduction experiment. Cells were incubated in the anoxic ICarb solution (20 mM PIPES, 2% NaCl, 5 mM sodium lactate, 10 mM Fe citrate and 50 mM NaHCO_3_). The collected samples were filtered at 0.22 µm and mixed in a 1:1 ratio with malachite green solution (0.7 M HCl, 0.3 mM malachite green oxalate, 8.3 mM Na_2_MoO_4_, 0.05% (v⁄v) Triton X-100). After 15 min of incubation at room temperature, the absorbance was measured at 630 nm and compared with values obtained with a standard curve prepared with 10 mM Na_2_HPO_4_^44^.

### Data availability statement

All data generated or analysed during this study are included in this published article (and its Supplementary Information files).

## Electronic supplementary material

Supplementary information
