# Supplementary material for: Bacterial iron reduction and biogenic mineral formation for the stabilisation of corroded iron objects
Source: Sci Rep. 2018 Jan 15;8:764. doi: 10.1038/s41598-017-19020-3 (PMC5768810; doi:10.1038/s41598-017-19020-3)
Supplement: Supplementary file 1 — Supplementary information [file 41598_2017_19020_MOESM1_ESM.doc]

**Supplementary information to the manuscript entitled**

*Bacterial iron reduction and biogenic mineral formation for the stabilisation of corroded iron objects*

**by**

Wafa M. Kooli^1,2^, Lucrezia Comensoli^1,2^, Julien Maillard^3^, Monica Albini^1,2^, Arnaud Gelb^3^, Pilar Junier^1*^ and Edith Joseph^2,4*^

^1^Laboratory of Microbiology, Institute of Biology, University of Neuchâtel, 2000 Neuchâtel, Switzerland, pilar.junier@unine.ch

^2^Laboratory of Technologies for Heritage Materials, Institute of Chemistry, University of Neuchâtel, Switzerland, edith.joseph@unine.ch

^3^Laboratory for Environmental Biotechnology, ENAC-IIE-LBE, Ecole Polytechnique Fédérale de Lausanne, 1015 Lausanne, Switzerland

^4^Haute Ecole Arc Conservation-Restauration, HES-SO, 2000 Neuchâtel, Switzerland, edith.joseph@he-arc.ch

*co-corresponding authors


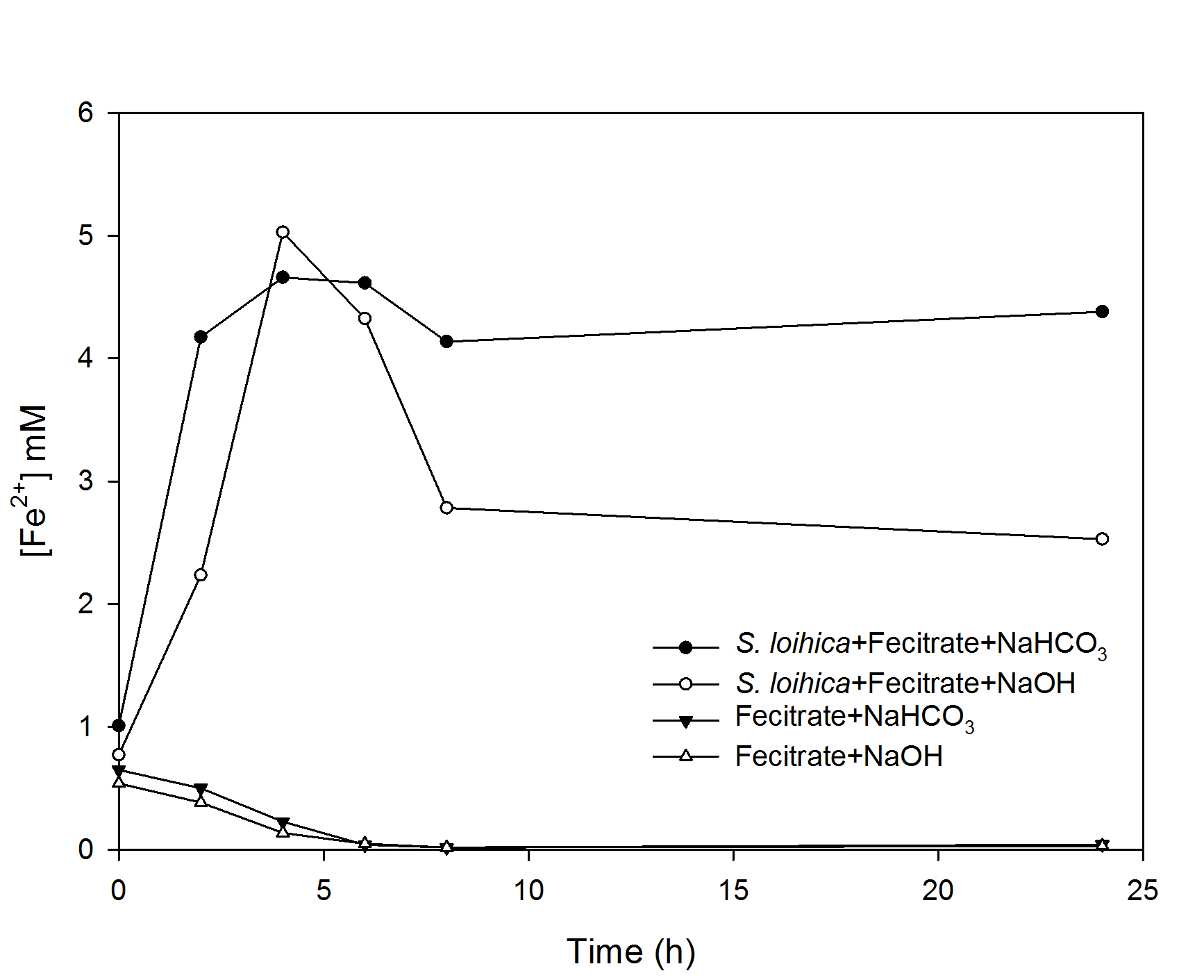

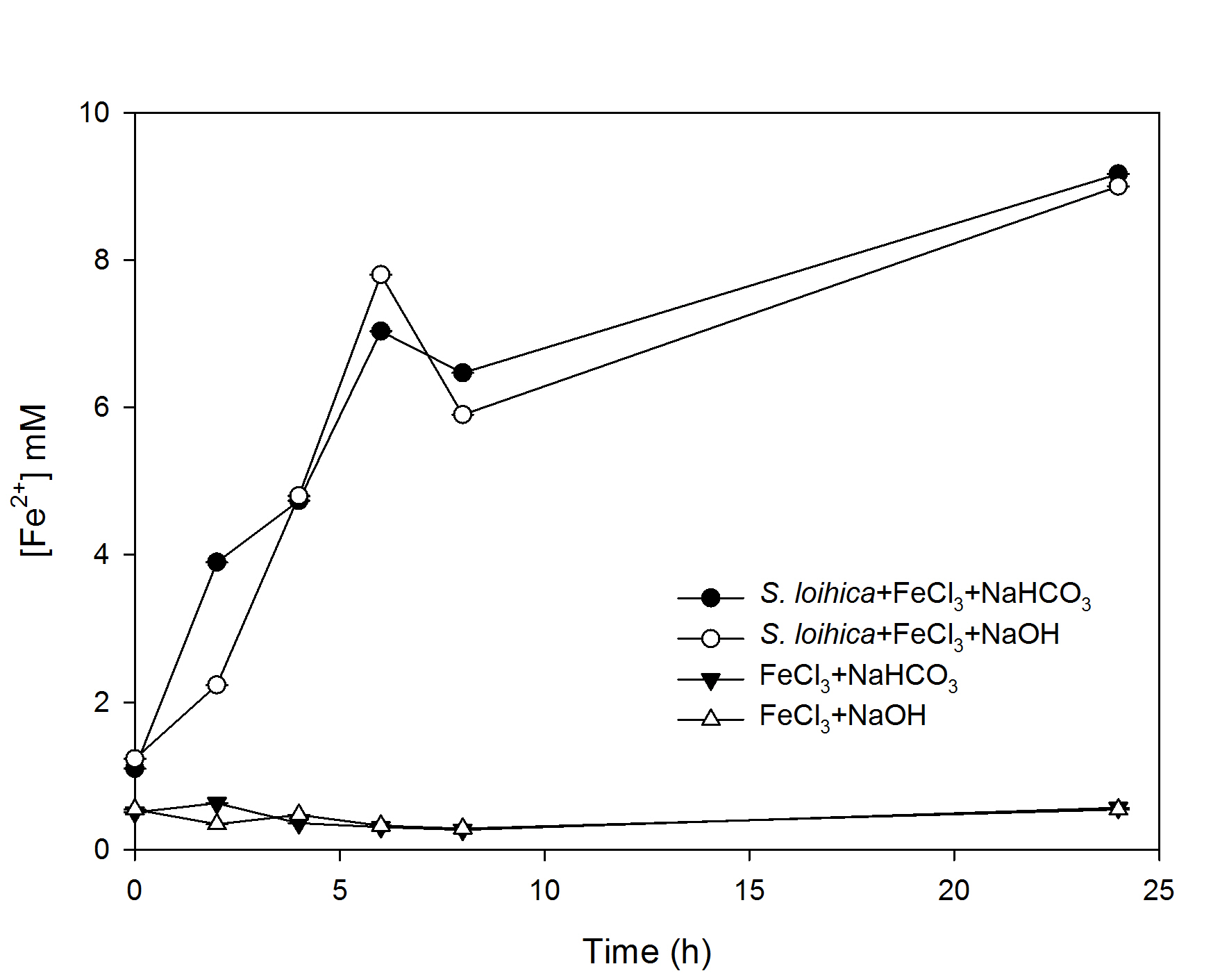


**Figure SI-1: Soluble Fe(III) reduction**. Evolution of Fe^2+^ concentration in the chemical matrices favouring the production of iron oxides (IOx, addition of NaOH) or iron carbonates (ICarb, addition of NaHCO_3_). The error bars correspond to the standard deviation of three independent measurements. These bars were however too small to be visible in the graphs. The presence of Fe^2+^ in the abiotic controls with Fe(III) citrate was investigated and it is explained by the contamination of the Fe(III) citrate product with Fe^2+^ (manufacturer contamination).

**
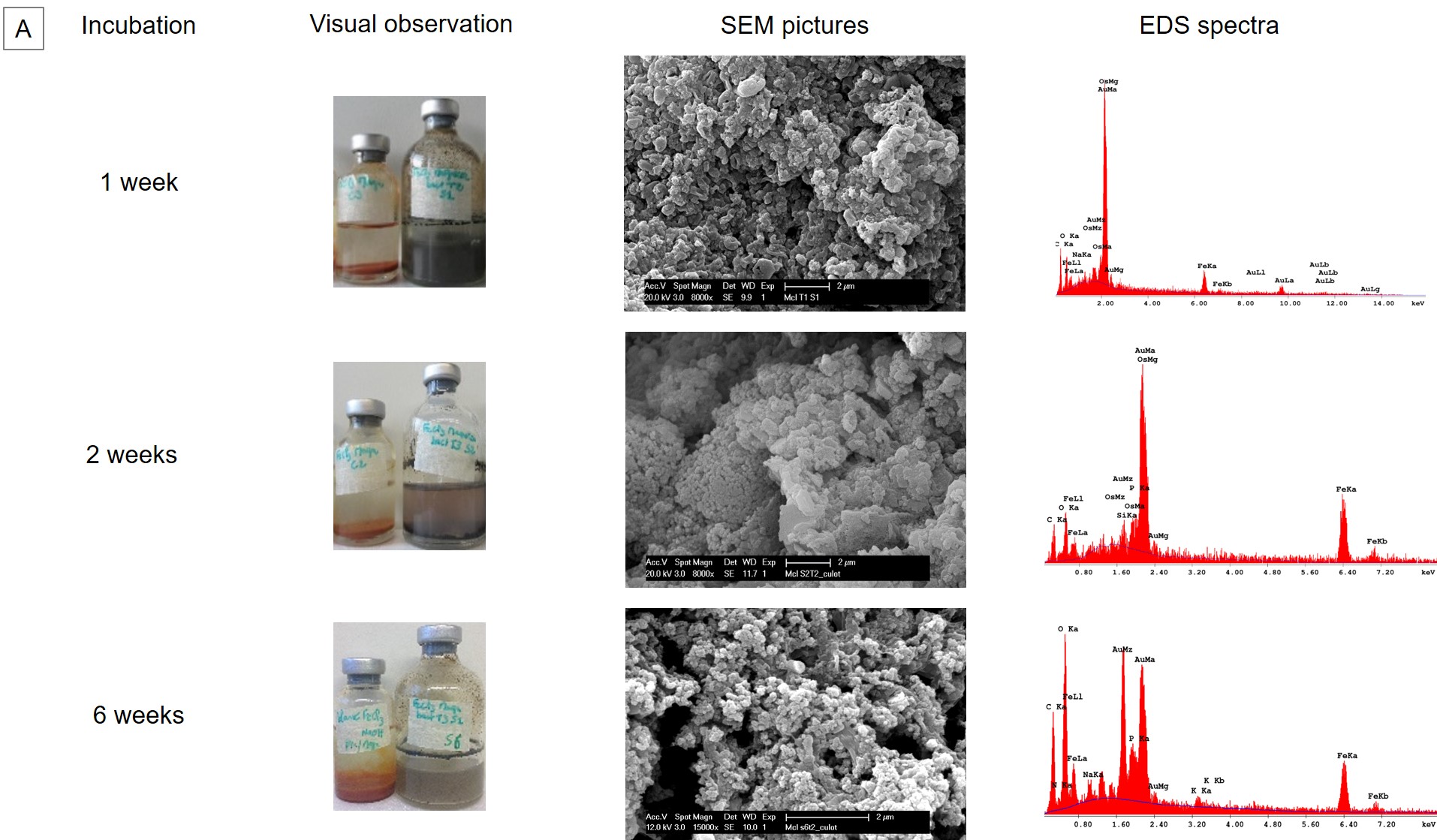
**

**
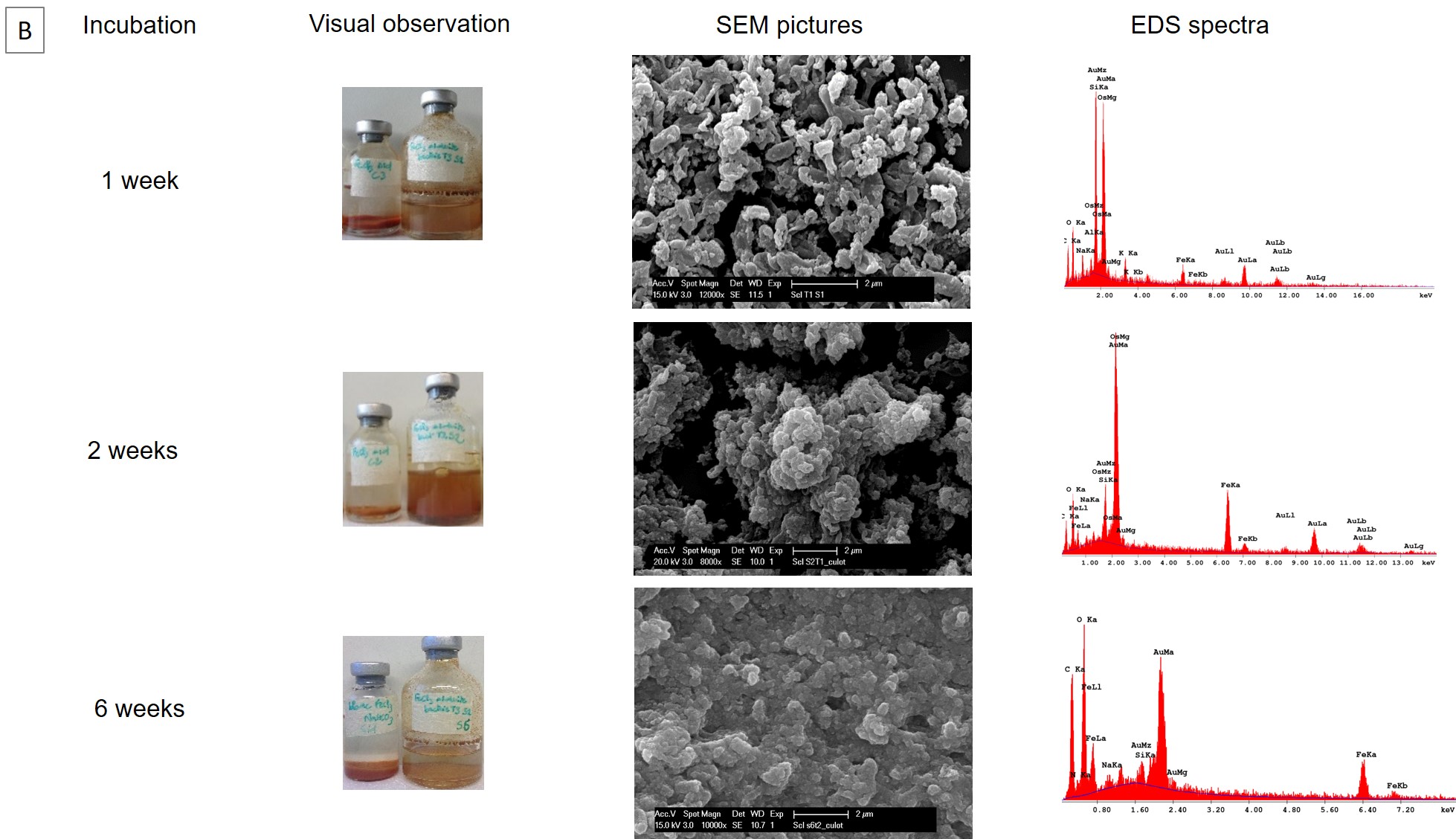
**

**
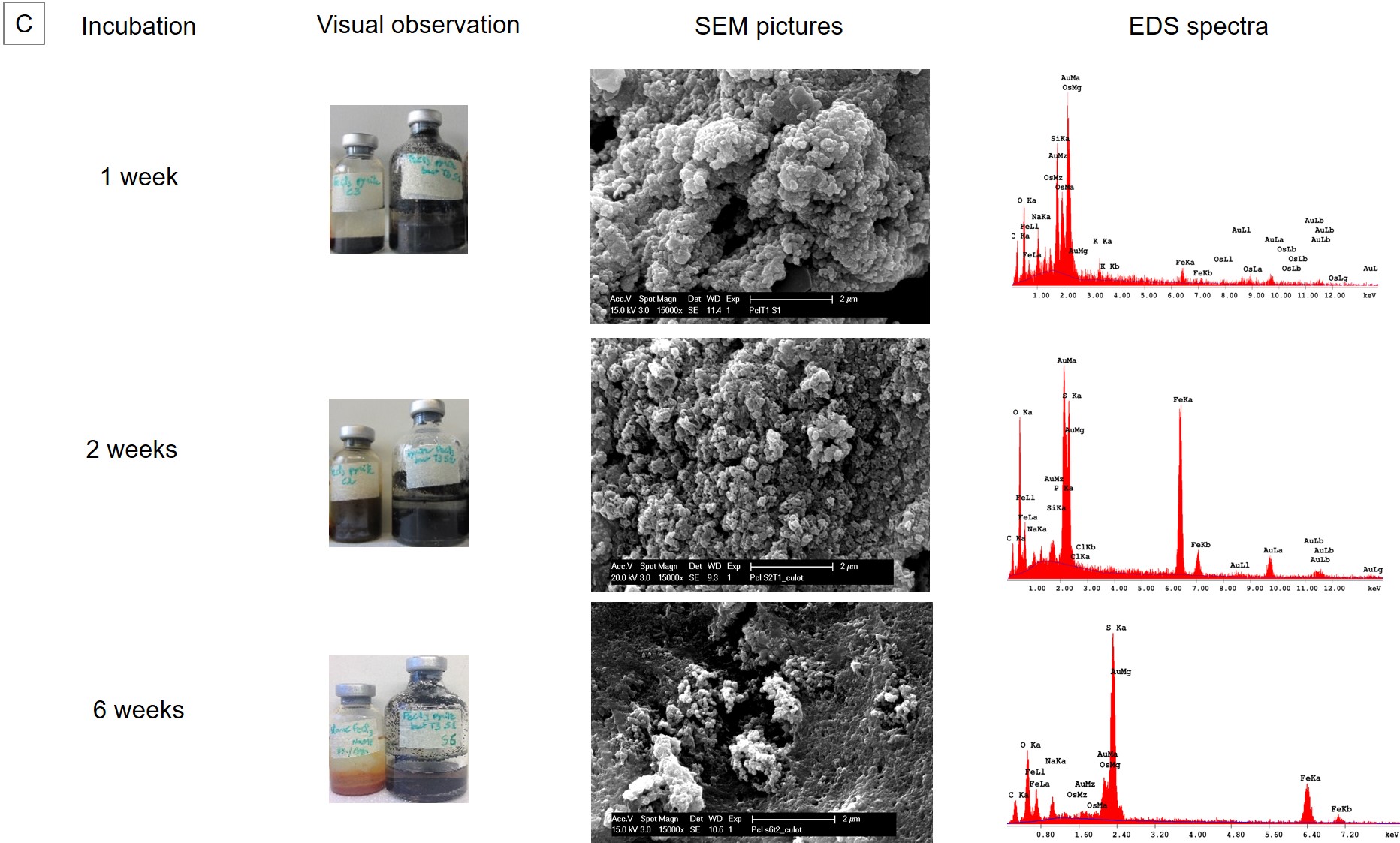
**


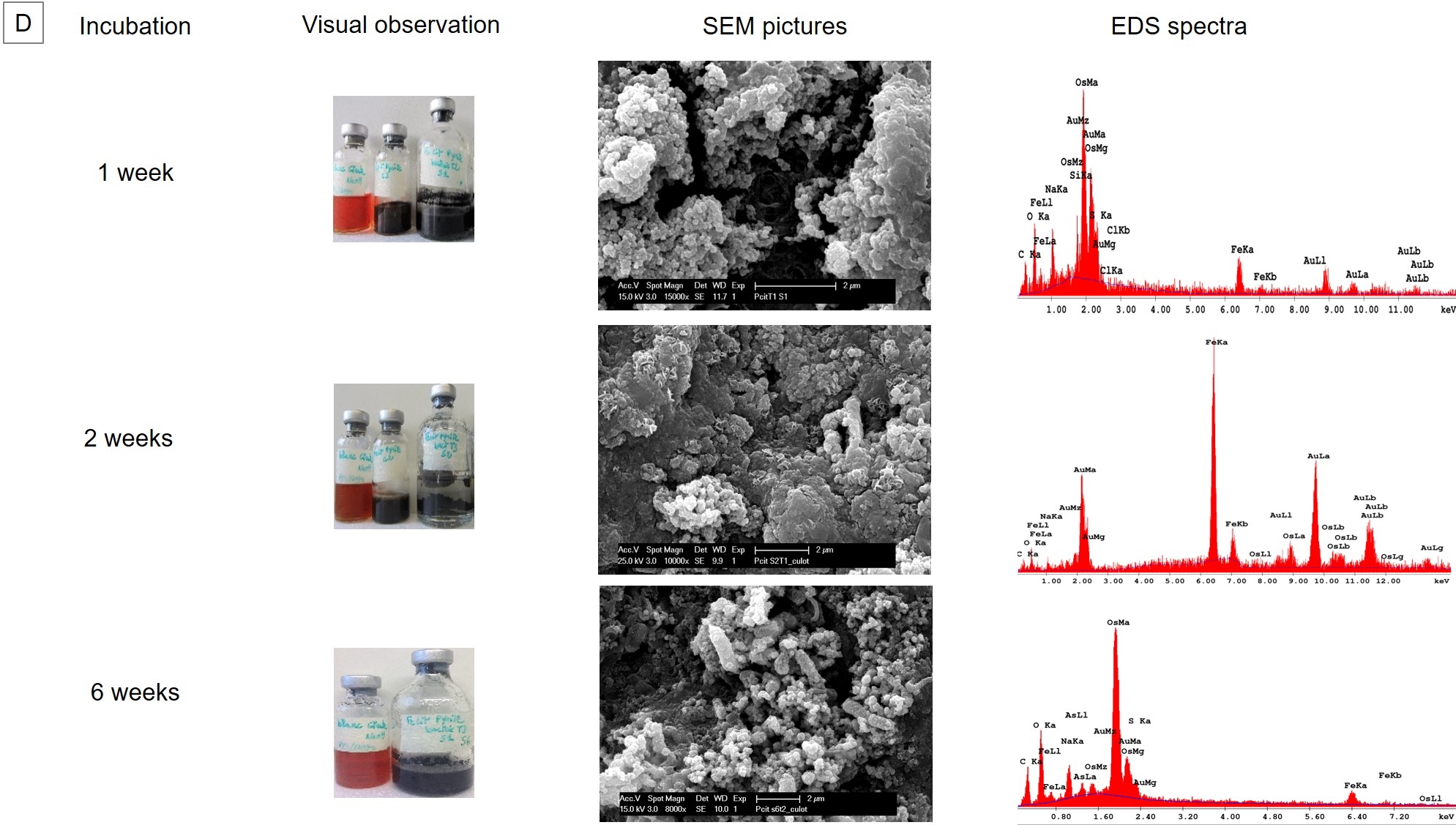
**Figure SI-2**: **Soluble Fe(III) reduction and biogenic mineral formation.** Visual observations, scanning electron microscopy (SEM) images and EDS spectra of culture pellets of *S. loihica*, after 1, 2 and 6 weeks of incubation under the (A) IOx, (B) ICard and (C) ISulf conditions with FeCl_3_, and (D) ISulp condition with Fe citrate.

**Table SI-3: Elemental composition in terms of atomic percentage (AT%) obtained using energy-dispersive X-ray spectroscopy (EDS), for the treatments using Fe citrate, which resulted in the formation of biogenic minerals (IOx and ICarb conditions).**

| E  L  E  M  E  N  T  S  (AT%) | 1 week | | | | 2 weeks | | | | 6 weeks | | | |
| --- | --- | --- | --- | --- | --- | --- | --- | --- | --- | --- | --- | --- |
|  | IOx conditions | | ICarbconditions | | IOx conditions | | ICarb conditions | | IOx conditions | | ICarb conditions | |
|  | + | - | + | - | + | - | + | - | + | - | + | - |
| Fe | 1.97 | 2.51 | **15.37** | 2.11 | 2.09 | - | **15.26** | - | **15.61** | - | **19.18** | - |
| O | 22.30 | 10.65 | 24.00 | - | 9.86 | - | 33.66 | 8.90 | 51.02 | 5.50 | 46.42 | 14.29 |
| C | 35.25 | 33.48 | 33.74 | 33.01 | 67.15 | - | 26.64 | 30.52 | 20.75 | 30.00 | 19.22 | 30.79 |
| P | **-** | **-** | **5.60** | **-** | **-** | **-** | **9.89** | **-** | **11.23** | **-** | **12.77** | **-** |
| Na | 3.24 | 24.86 | 2.16 | 33.19 | - | 7.74 | 0.81 | 23.17 | - | 28.88 | - | 24.56 |
| Si | 9.57 | - | 3.11 | - | - | - | 1.34 | - | - | - | - | - |
| Os | 1.08 | - | 2.56 | - | 2.02 | - | 1.45 | - | - | 0.04 | - | - |
| Al |  | - | 0.62 | - | - | - | - | - | - | - | - | - |
| K | 1.18 | - | - | - | 0.37 | - | - | - | - | - | - | - |
| Cl | - | 25.17 | - | 29.56 | - | 68.91 | - | 23.82 | - | 31.50 | - | 27.43 |
| N | - | - | - | - | - | - | 4.28 | 6.47 | - | - | - | - |
| Au | 25.42 | 3.34 | 12.83 | 2.13 | 18.51 | 23.35 | 6.69 | 7.13 | 1.40 | 4.09 | 2.41 | 2.93 |

**
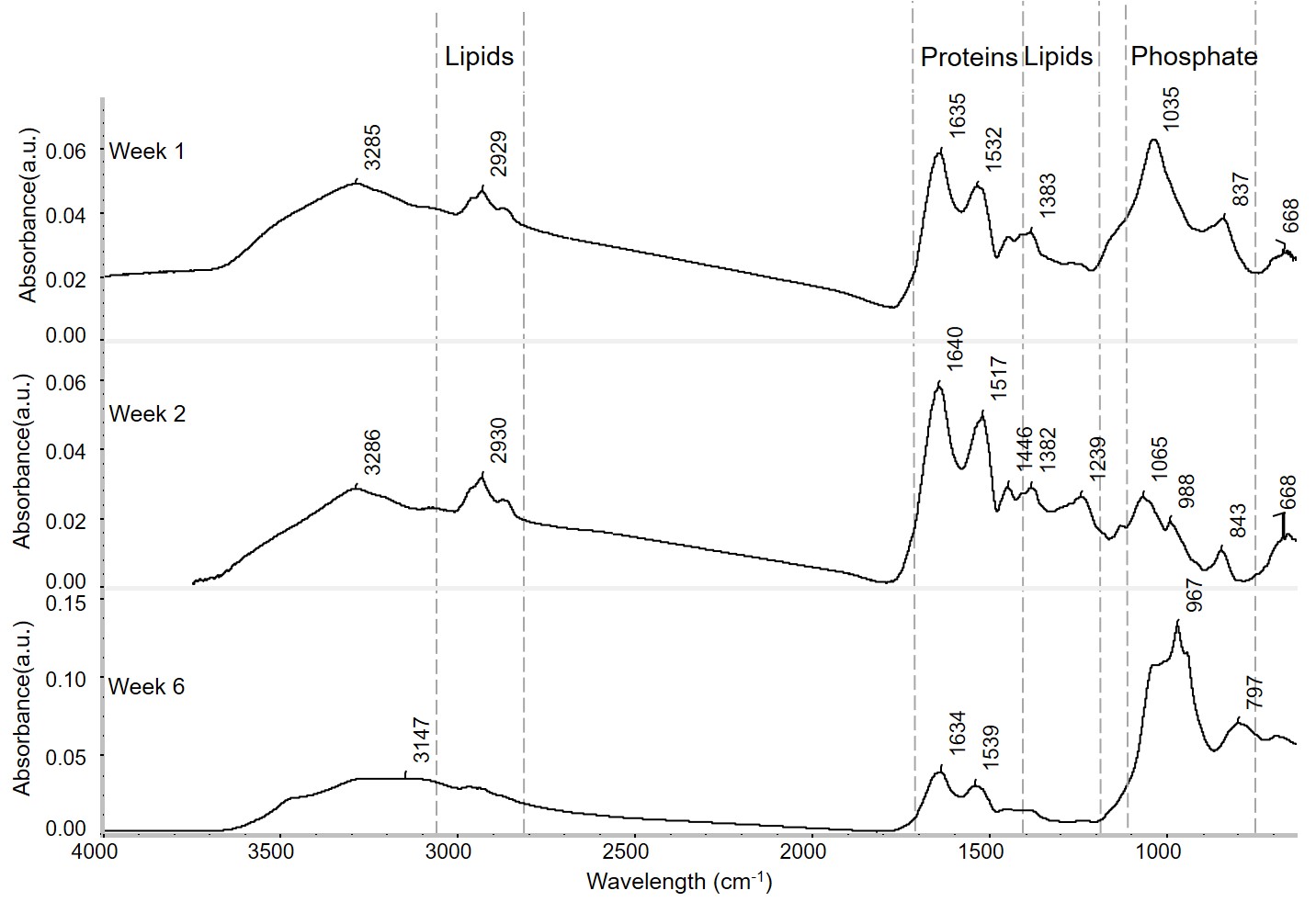
**

**Figure SI-4**: ATR-FTIR spectra of culture pellets of *Shewanella* *loihica* after 1, 2 and 6 weeks of incubation in Fe citrate-amended solution under IOx condition. The region corresponding to the absorbance peaks of lipids, proteins, and phosphates are flanked by dashed lines as indicated in the graph.


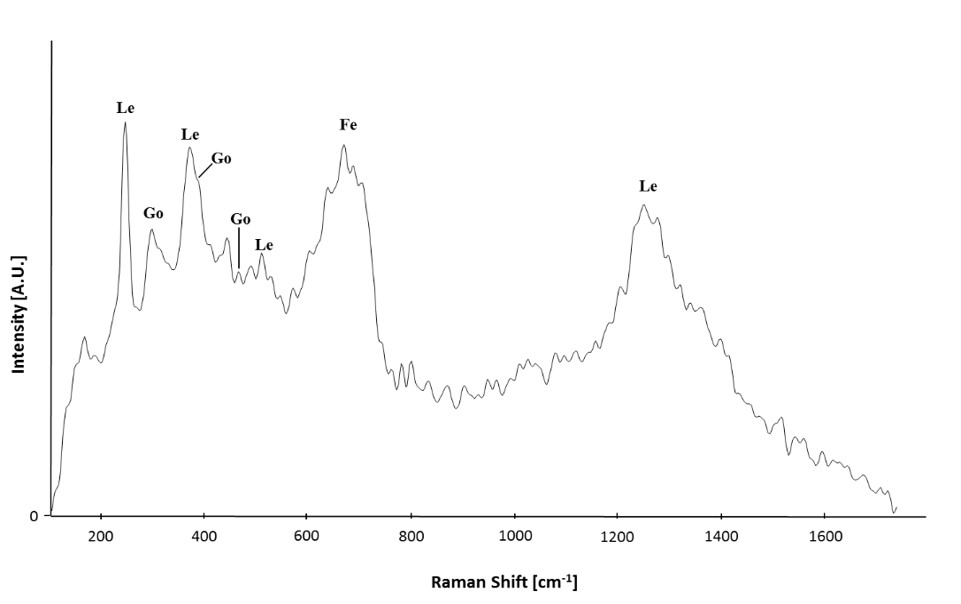


**Figure SI-5. Raman spectrum of the untreated iron coupon presenting a marine atmospheric corrosion.** Corrosion compounds were identified as lepidocrocite (Le), goethite (Go), and ferrihydrite (Fe).

**
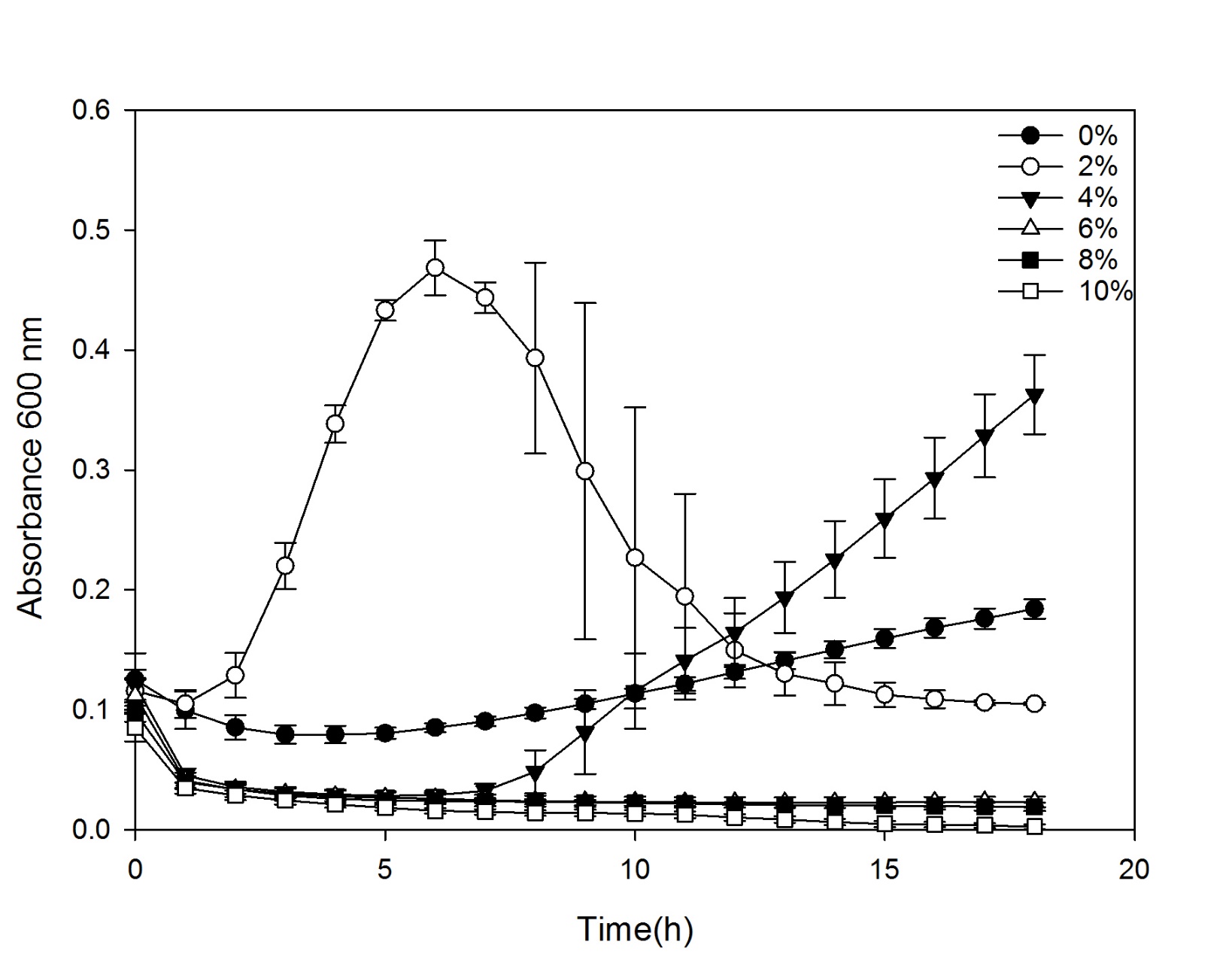
**

**Figure SI-6:** Evaluation of the growth of *S. loihica* at different NaCl concentrations using modified Tryptic Soy Broth medium (composition: 0.25% glucose, 0.25% K_2_HPO_4_, 0.3% soy peptone and 1.7% casein digest, pH7) with different NaCl concentrations (0, 2, 4, 6, 8 or 10% (w/v). Growth was monitored under agitation at room temperature measuring the absorbance at 600 nm for 18 hours.

**
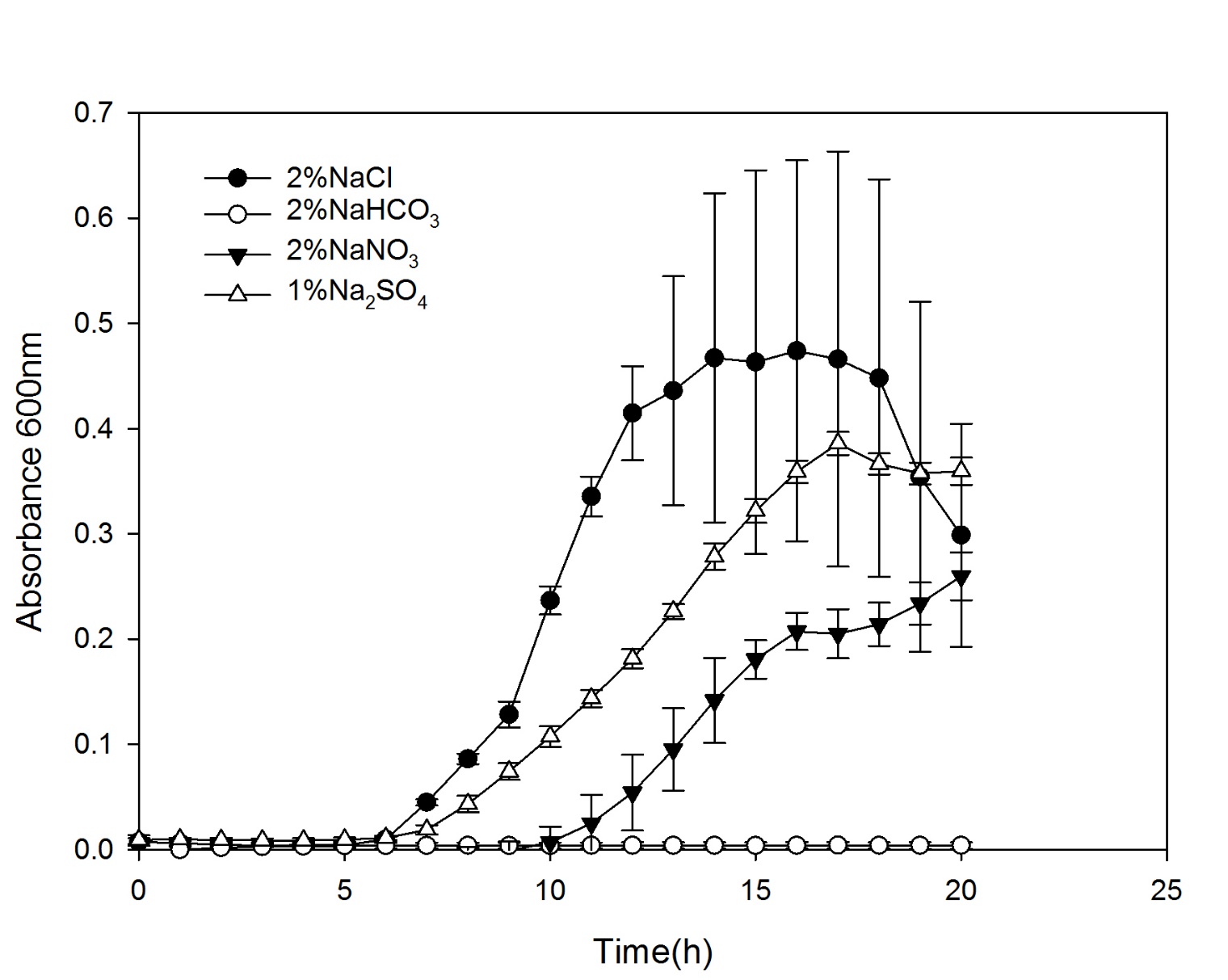
**

**Figure SI-7**: Evaluation of the chlorides dependence of *S. loihica* growth*.* The test was performed using modified Tryptic Soy Broth medium (composition: 0.25% glucose, 0.25% K_2_HPO_4_, 0.3% soy peptone and 1.7% casein digest, pH7) with different sodium sources (2% NaCl, 2% NaHCO_3_, 2% NaNO_3_ or 1% Na_2_SO_4_). Growth was monitored under agitation at room temperature, measuring the absorbance at 600 nm for 20 hours.


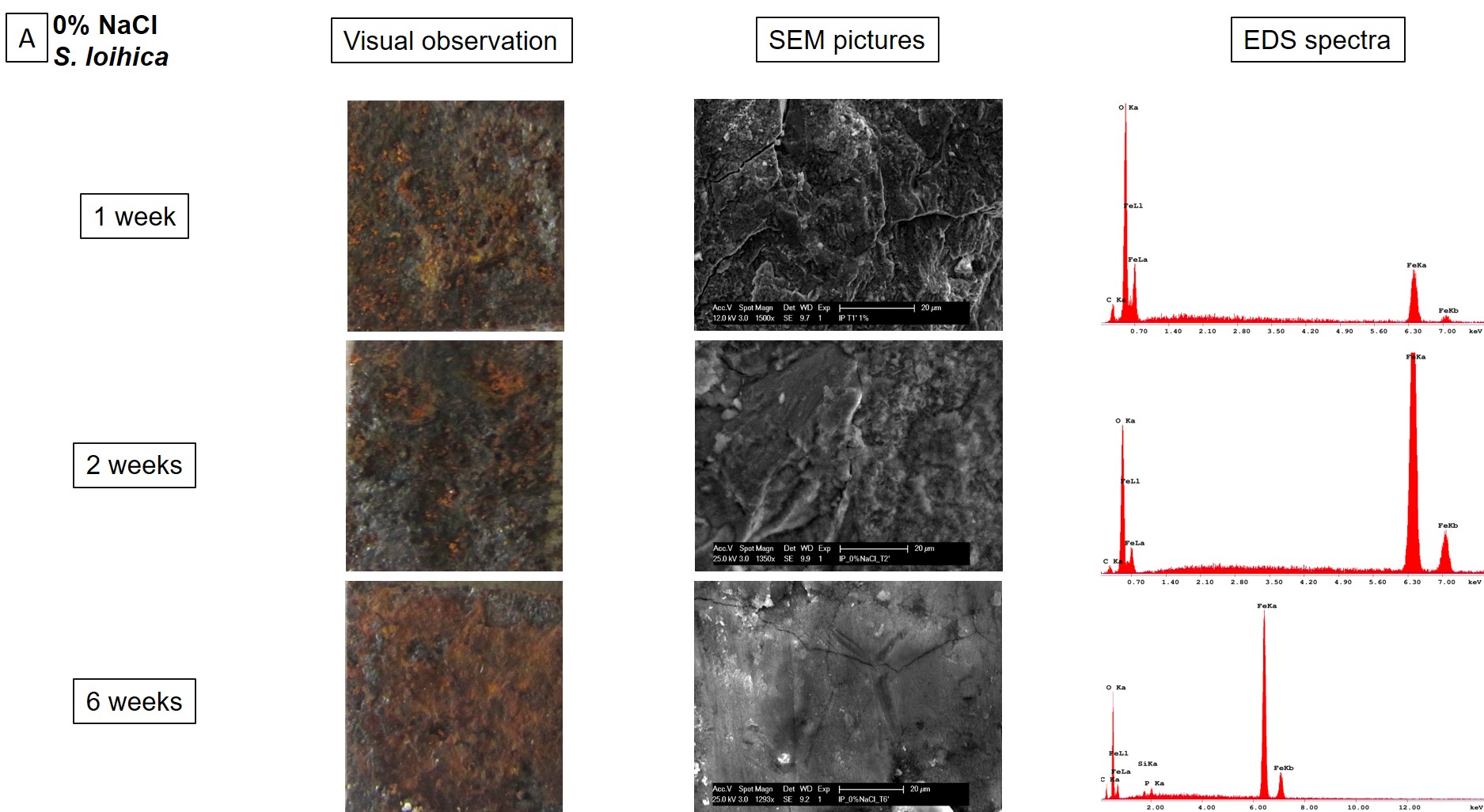


**
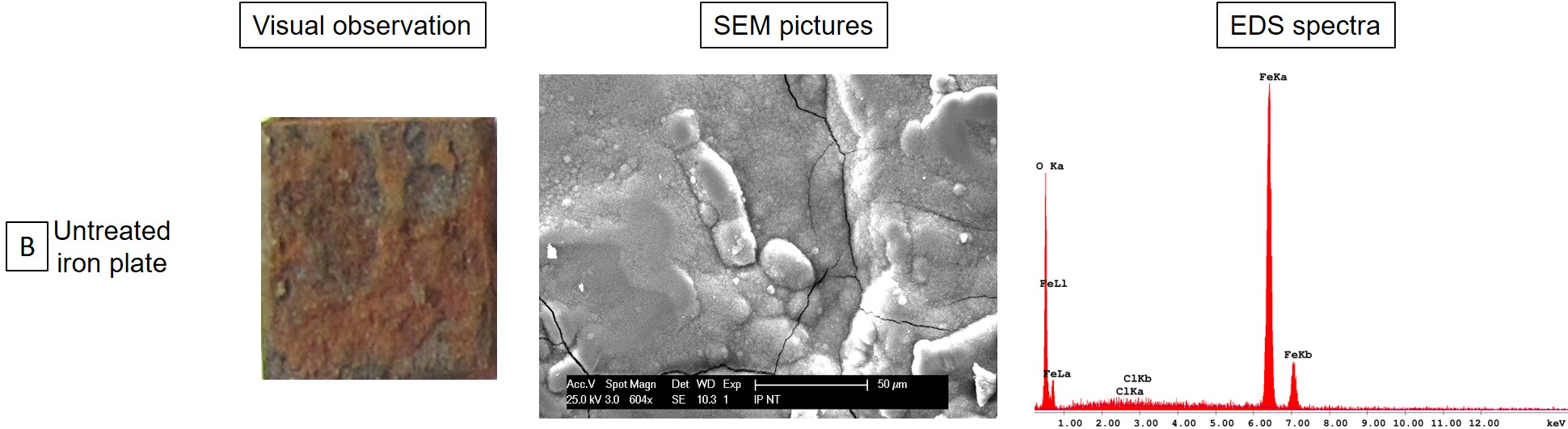
**

**Figure SI-8**: **Solid Fe(III) reduction and biogenic mineral formation.** Visual observations, scanning electron microscopy (SEM) images and EDS spectra of (A) the iron coupons treated with *S.* *loihica* at 0% NaCl under ICarb condition after incubation of 1, 2 and 6 weeks and B) the untreated control iron coupon.

**Table SI-9:** Atomic percentages (AT%) of the elements obtained from the Energy-dispersive X-ray spectroscopy (EDS) measurements performed on the iron coupons after 1, 2 and 6 weeks of incubation at 0% NaCl (+), on the abiotic control (-) and on the untreated coupon.

| Elements (AT%) | 1 week | | 2 weeks | | 6 weeks | | Untreated iron coupon |
| --- | --- | --- | --- | --- | --- | --- | --- |
|  | + | - | + | - | + | - |  |
| Fe | 45.31 | 53.80 | 42.00 | 58.41 | 29.50 | 48.63 | 27.20 |
| O | 40.08 | 46.20 | 43.07 | 41.59 | 37.27 | 47.42 | 50.56 |
| C | 14.62 | - | 14.93 | - | 32.25 | - | 19.68 |
| P | - | - | - | - | 0.98 | - | - |
| Na | - | - | - | - | - | - | - |
| Si | - | - | - | - | - | 3.95 | 1.04 |
| Cl | - | - | - | - | - | - | 1.53 |


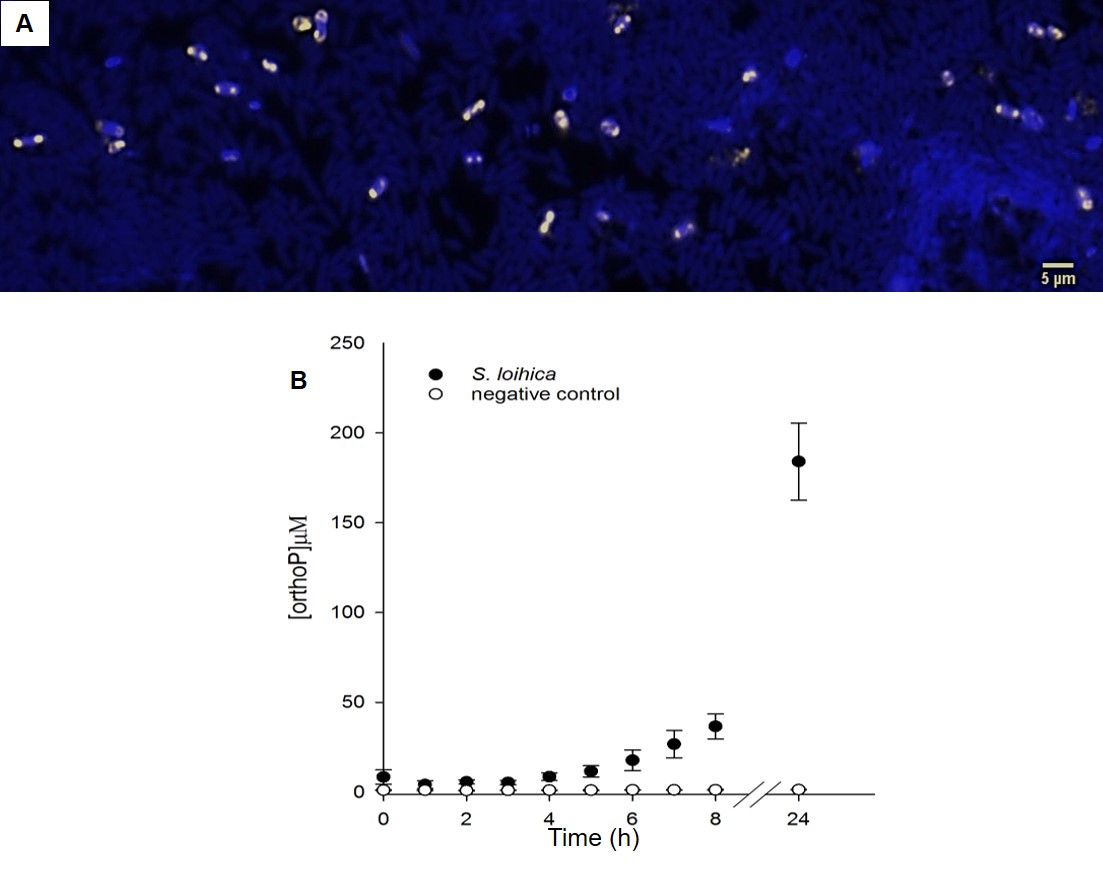
**Figure SI-10. Evaluation of polyphosphates accumulation and orthophosphates release by *S. loihica*.** (A) Epifluorescence microscopy image showing polyphosphate granules (yellow spots) in the cells of *S. loihica* revealed by DAPI staining. (B) Evolution of orthophosphates concentration in the supernatant of *S. loihica* cultures incubated in anoxic ICarb solution.


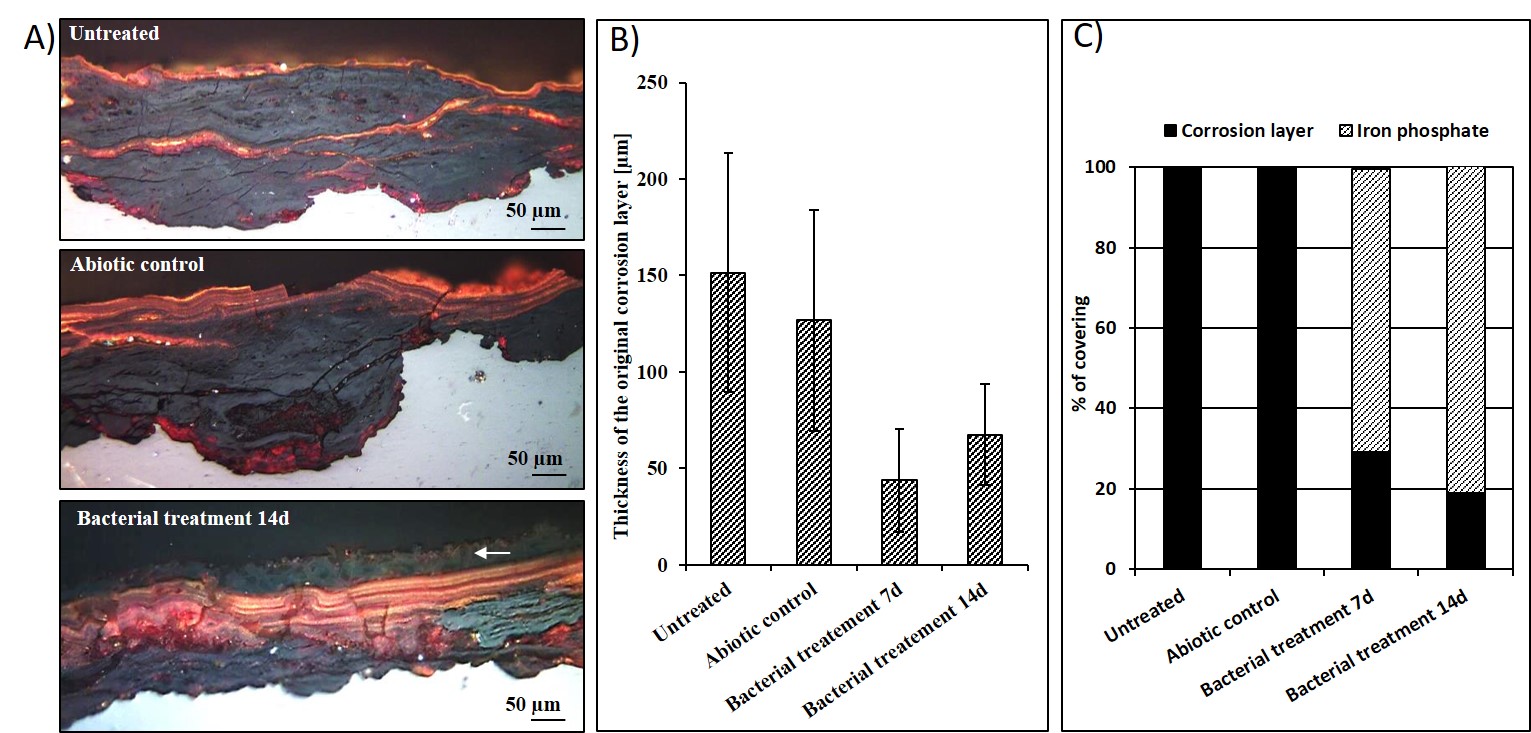


**Figure SI-11. Effect of biogenic mineral formation on the corrosion layer of iron coupons.**A) Microscopic images of the untreated, abiotic control, and bacteria-treated iron coupons after 2 weeks of incubation. B) Measurement of the thickness of the original corrosion layer. C) Estimation of the coverage of the original corrosion layer by newly formed iron phosphates. 7d and 14d correspond to the treatment after 1 and 2 weeks respectively. The white arrow indicates the grey-black layer of iron minerals formed after 2 weeks of treatment.


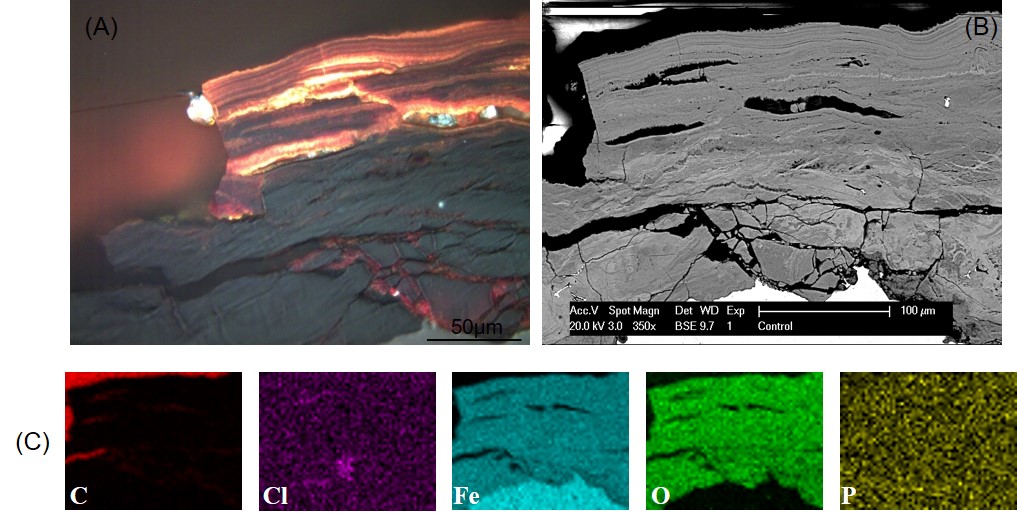


**Figure SI-12**. **Cross-section analysis of the corrosion layer in abiotic iron coupons**. (A) Optical Microscopy and (B) scanning electron microscopy (SEM) images of the abiotic coupon after 6 weeks of incubation. (C) Elemental mapping showing the presence of carbon (red), chlorine (purple), iron (blue), oxygen (green) and phosphorus (yellow).


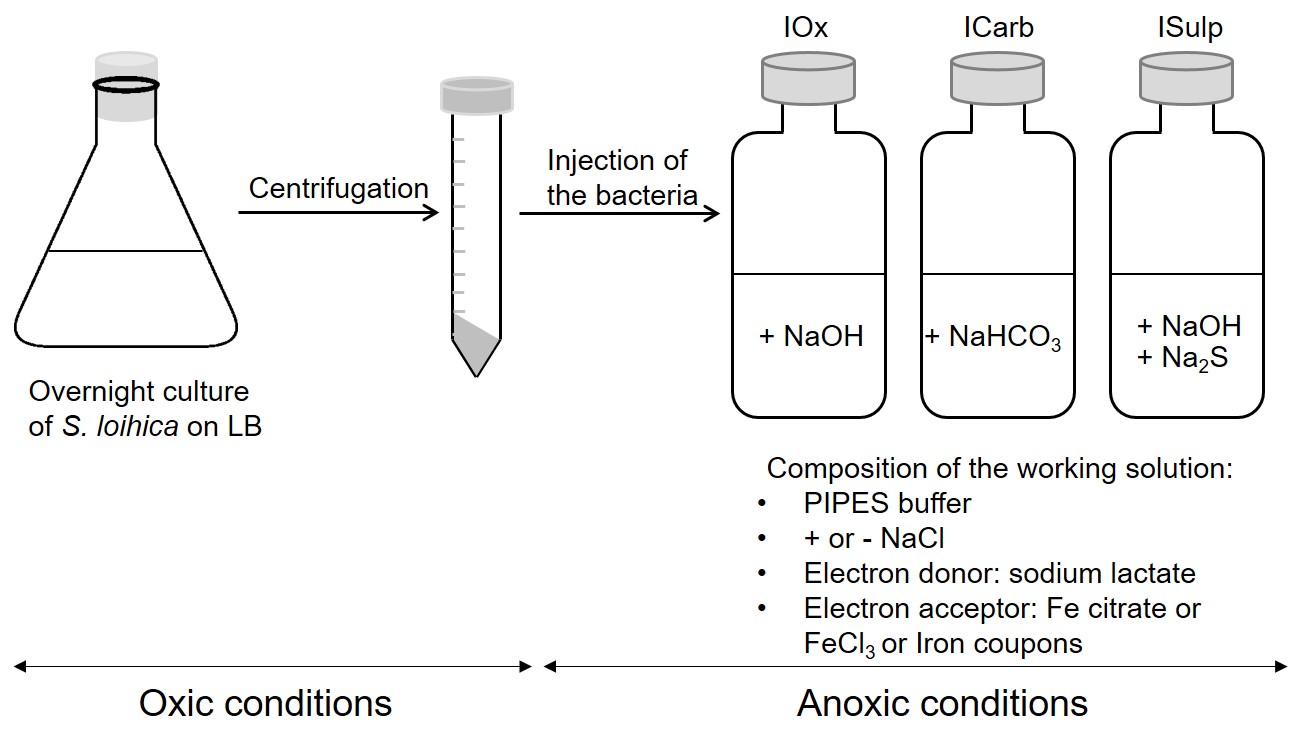


**Figure SI-13**: Schematic representation of the procedure used to promote iron reduction and biogenic mineral formation by *S. loihica* in chemically controlled conditions. Iron coupons were only used with ICarb condition.
